# Ancestral lysosomal enzymes with increased activity harbor therapeutic potential for treatment of Hunter syndrome

**DOI:** 10.1016/j.isci.2021.102154

**Published:** 2021-02-06

**Authors:** Natalie M. Hendrikse, Anna Sandegren, Tommy Andersson, Jenny Blomqvist, Åsa Makower, Dominik Possner, Chao Su, Niklas Thalén, Agneta Tjernberg, Ulrica Westermark, Johan Rockberg, Stefan Svensson Gelius, Per-Olof Syrén, Erik Nordling

**Affiliations:** 1 Swedish Orphan Biovitrum AB, Stockholm 112 76, Sweden; 2 Science for Life Laboratory, School of Engineering Sciences in Chemistry, Biotechnology and Health, KTH Royal Institute of Technology, Solna 171 21, Sweden; 3 Department of Fibre and Polymer Technology, School of Engineering Sciences in Chemistry, Biotechnology and Health, KTH Royal Institute of Technology, Stockholm 100 44, Sweden; 4 Department of Protein Science, School of Engineering Sciences in Chemistry, Biotechnology and Health, KTH Royal Institute of Technology, Stockholm 10691, Sweden

**Keywords:** Biochemistry, Structural Biology

## Abstract

We show the successful application of ancestral sequence reconstruction to enhance the activity of iduronate-2-sulfatase (IDS), thereby increasing its therapeutic potential for the treatment of Hunter syndrome—a lysosomal storage disease caused by impaired function of IDS. Current treatment, enzyme replacement therapy with recombinant human IDS, does not alleviate all symptoms, and an unmet medical need remains. We reconstructed putative ancestral sequences of mammalian IDS and compared them with extant IDS. Some ancestral variants displayed up to 2-fold higher activity than human IDS in *in vitro* assays and cleared more substrate in *ex vivo* experiments in patient fibroblasts. This could potentially allow for lower dosage or enhanced therapeutic effect in enzyme replacement therapy, thereby improving treatment outcomes and cost efficiency, as well as reducing treatment burden. In summary, we showed that ancestral sequence reconstruction can be applied to lysosomal enzymes that function in concert with modern enzymes and receptors in cells.

## Highlights

• Reconstruction of ancestral lysosomal enzymes that function in complex cellular context
• Ancestral iduronate-2-sulfatases with increased activity compared with the human enzyme
• Increased clearance of substrate in patient fibroblasts indicates therapeutic potential

## Graphical abstract

**Figure:**
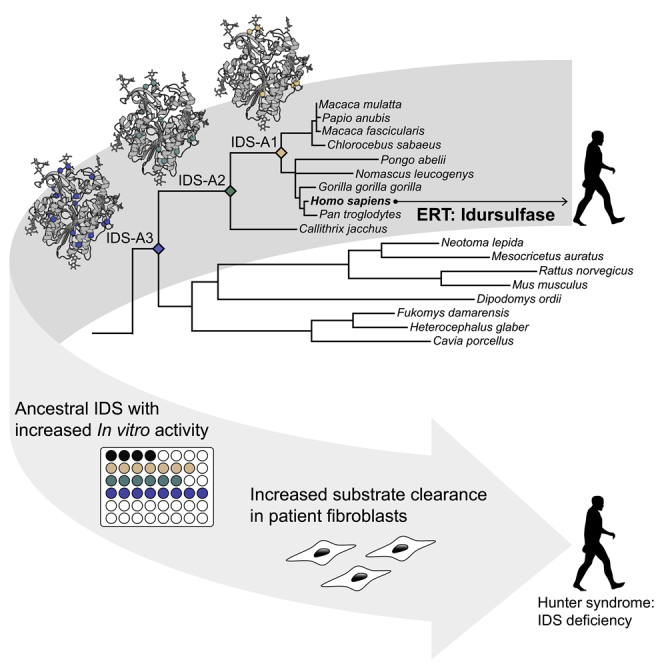

## Introduction

Lysosomal storage diseases (LSDs) are a group of more than 50 known hereditary disorders that are associated with impaired or depleted function of one or several lysosomal enzymes (Boustany, 2013). The most common treatment today is enzyme replacement therapy (ERT). As of 2020, ERT has been approved by the Food and Drug Administration for nine LSDs (Chen et al., 2019), but dosage and distribution remain challenging and do often not alleviate all symptoms. Required doses are generally high, resulting in lengthy intravenous administration and costly production of the drugs (Desnick and Schuchman, 2012; Grabowski and Whitley, 2017). Increasing stability and activity of therapeutic enzymes is therefore of great interest, as both properties are directly proportional to the required dose to achieve the desired therapeutic effect. To advance ERT for LSDs, the design of novel enzyme variants with improved properties is actively investigated. Previously, the catalytic efficiency of arylsulfatase A—the enzyme associated with LSD metachromatic leukodystrophy—was enhanced up to 5-fold by just a few amino acid substitutions that occurred in the evolution of non-human mammals (Simonis et al., 2019), inspired by the observation that arylsulfatase A from mouse displayed higher activity than its human homolog. Phylogenetic analyses have also been used to study the evolution of the phosphatase superfamily, which also includes arylsulfatases (Van Loo et al., 2019). These findings encouraged us to explore ancestral sequence reconstruction (ASR) (Gumulya and Gillam, 2017; Merkl and Sterner, 2016; Risso and Sanchez-Ruiz, 2017; Thornton, 2004; Wilding et al., 2017) to engineer lysosomal sulfatases. ASR is a bioinformatics method that has yielded valuable insight into enzyme evolution (Nguyen et al., 2016; Risso et al., 2013) and protein structure-function relationships (Nicoll et al., 2020; Schupfner et al., 2020; Wilson et al., 2015), but has rarely been applied in the context of biopharmaceuticals. One exception is the engineering of coagulation factor VIII with improved expression rates and decreased decay rates, allowing for lower doses in gene therapy experiments toward treatment of hemophilia A (Samelson-Jones and Arruda, 2019; Zakas et al., 2017). We have previously applied ASR toward improving therapeutic properties of phenylalanine/tyrosine ammonia lyases and obtained enzyme scaffolds with increased thermal and long-term stability as well as altered substrate specificities (Hendrikse et al., 2020).

We aimed to explore the application of ASR to engineer lysosomal enzymes toward treatment of LSDs—a therapeutic area with a remaining significant unmet medical need (Desnick and Schuchman, 2012). To this end we chose to apply the method to iduronate-2-sulfatase (IDS, EC 3.1.6.13), a monomeric enzyme that catalyzes the first step of the lysosomal breakdown pathway of glycosaminoglycans (GAGs) heparan sulfate and dermatan sulfate (Figure S1A). Hunter syndrome, or mucopolysaccharidosis type II (MPS II, OMIM 309900), is an X-linked genetic LSD caused by impaired function of IDS, which leads to accumulation of these GAGs in lysosomes of tissues and organs, thereby causing cellular dysfunction and organ failure (Bach et al., 1973; Hunter, 1917; Wilson et al., 1990). To be catalytically active, IDS requires N-linked glycosylation and post-translational modification of a cysteine in the active site to formylglycine (fGly). The latter is performed by the formylglycine-generating enzyme (FGE, EC 1.8.3.7) (Von Figura et al., 1998; Schmidt et al., 1995), which converts the cysteine side chain (-CH_2_-SH) into an aldehyde group (-CH=O). Crystallization efforts revealed the likely presence of a calcium ion next to fGly in the active site, which is believed to stabilize the sulfate-ester formation and has also been observed in other human sulfatases (Demydchuk et al., 2017).

Over 500 pathogenic mutations in the *IDS* gene have been reported, distributed over more than 100 positions in the enzyme sequence, and structural information has provided valuable insight into the underlying disease-causing mechanisms (Demydchuk et al., 2017). Approximately two-thirds of patients with MPS II are affected by progressive deterioration of the central nervous system (CNS) associated with neuronal degradation, and patients with this severe, early-onset form of the disease seldom reach adulthood (Whiteman and Kimura, 2017). The current option for treatment is ERT with the recombinant human enzyme (Chen et al., 2019; Whiteman and Kimura, 2017), which is available in two different forms: idursulfase (Elaprase, Shire Pharmaceuticals/Takeda Pharmaceutical Company Ltd., approved for therapeutic usage in the United States in 2006 [Heartlein and Kimura, 2014; Muenzer et al., 2006]) and idursulfase beta [Hunterase, Green Cross Corporation, approved for therapeutic usage in South Korea in 2012 [Kim et al., 2017; Sohn et al., 2013]). Both enzymes are effective in treating somatic symptoms of the disease and reducing urinary excretion of GAGs (Kim et al., 2017), but have no documented impact on CNS manifestations as they do not achieve therapeutic levels of activity in the brain.

We selected human IDS (hIDS) and murine IDS (mIDS) as reference enzymes and compared them with several ancestral IDS enzymes, going back from the hIDS sequence to the last common ancestor of primates and rodents. Owing to the high degree of conservation of IDS in mammals, the oldest ancestral variant contained only 20 substitutions when compared with the extant hIDS. We found that some ancestral enzymes displayed increased activity *in vitro* when compared with hIDS, mIDS, and idursulfase, which was also evident in the fibroblasts of patients with MPS II. This increased activity may allow for lower dosage or enable enzyme levels that achieve a therapeutic effect in the brain. We showed that ASR can be successfully applied to enzymes that function intracellularly and in concert with modern enzymes and receptors, which may open up doors for various *in vivo* applications.

## Results

### Reconstructed ancestral IDS enzymes are highly conserved and can be functionally expressed

**Figure 1:**
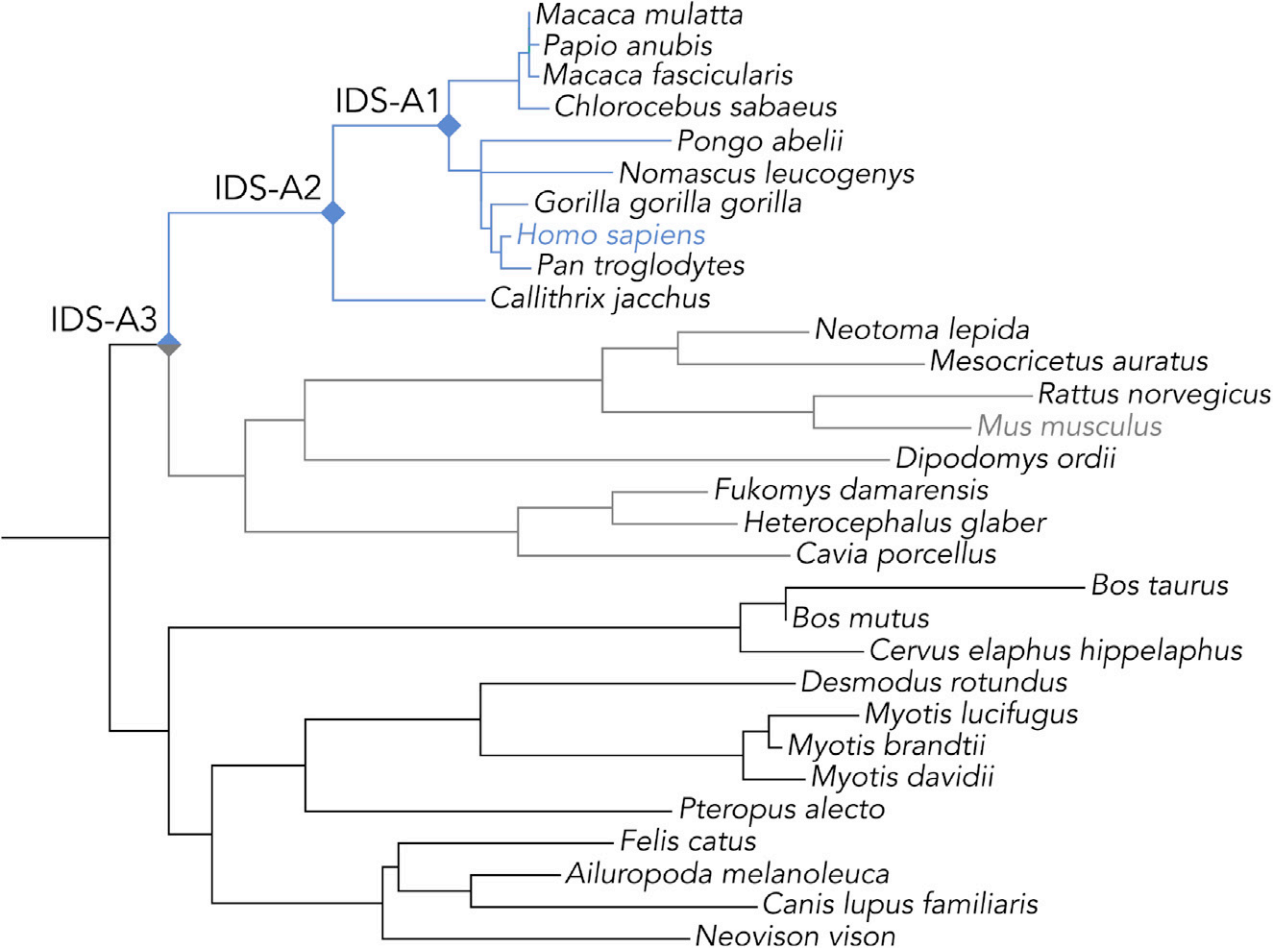
Phylogenetic tree of mammalian IDS homologs Maximum likelihood tree of 30 mammalian IDS sequences. The colored clades are primates (blue) and rodents (gray), and reference enzymes hIDS and mIDS are colored accordingly. The full sequence alignment and phylogenetic tree can be found in Figure S2.

**Figure 2:**
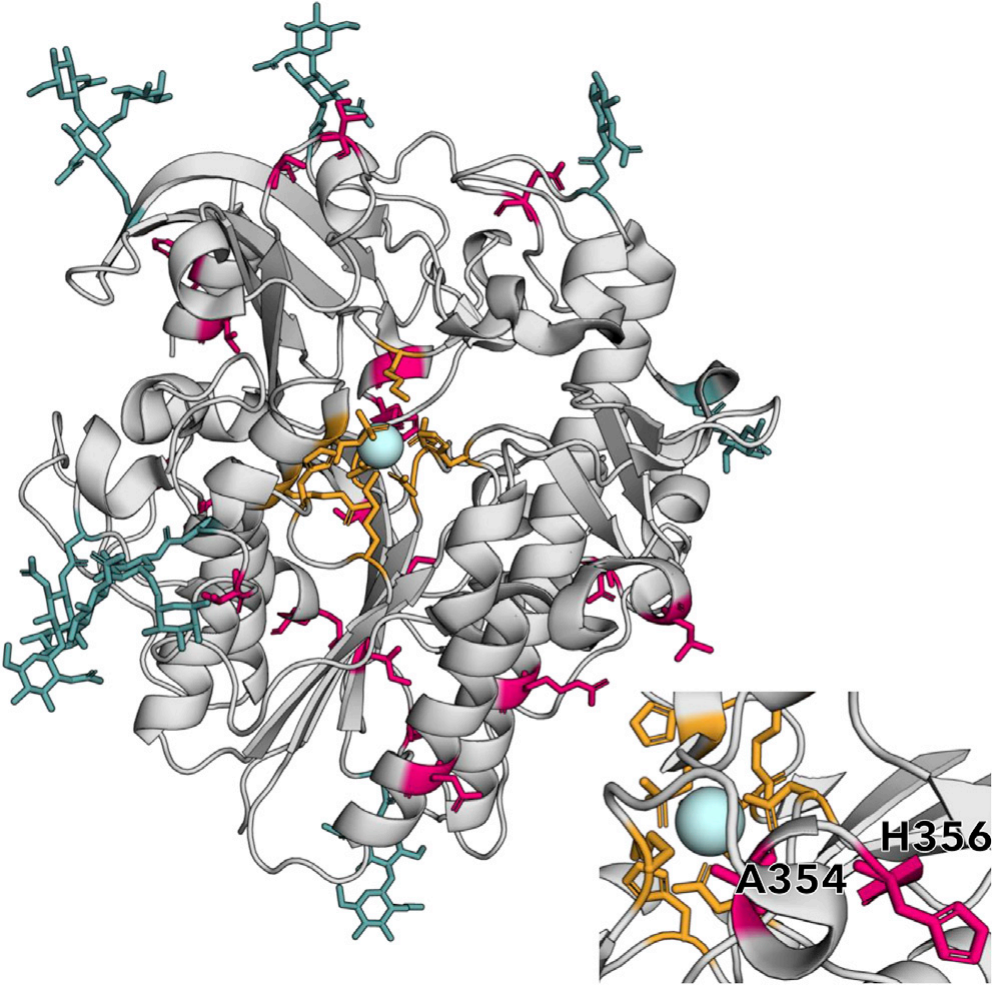
Positions substituted in IDS-A3 shown on the structure of hIDS All 20 residues that are different in IDS-A3 are colored in pink in the structure of hIDS (PDB: 5FQL (Demydchuk et al., 2017)). Residues in the active site are shown as orange sticks, glycans are shown as cyan sticks, and the calcium ion in the active site is shown as a light blue sphere. The insert shows a close-up of the active site and the location of residues A354 and H356. The figure was prepared using PyMOL version 2.4 (The PyMOL Molecular Graphics System, Version 2.0 Schrödinger, LLC). See also Figure S3 and Table S1.

The human IDS sequence was used as a query for constructing a phylogenetic tree (Figure 1, a complete sequence alignment and full tree with accession numbers and bootstrap values can be found in Figures S2A and S2B, respectively). We used maximum likelihood statistics implemented in PAML (Yang, 1997, 2007) to reconstruct the most likely ancestral sequences at three nodes between hIDS and the last common ancestor of primates and rodents. Owing to the high degree of conservation of IDS in mammals, the putative ancestral sequences had the same length as hIDS and only 6, 12, and 20 amino acid substitutions were predicted for the IDS-A1, IDS-A2, and IDS-A3, respectively (Figure S3). All positions at which different residues were predicted in the ancestors (referred to as *ancestral mutations*) are highlighted on the structure of hIDS in Figure 2. None of the predicted mutations occurred in one of the 128 positions where disease-causing mutations have been reported (Demydchuk et al., 2017). Inspection of the reconstructed sequences showed that cysteine 84, which is post-translationally modified to fGly, as well as all known catalytic residues were conserved (Figure S3). All glycosylation sites were also conserved, apart from a threonine to serine mutation in the motif around N144 for IDS-A2 and IDS-A3. Only two changes occurred relatively close to catalytic residues (see close-up in Figure 2): mutation A354T in IDS-A3 is located at 4.9 Å from K347 and mutation H356R in IDS-A2 and IDS-A3 is located 9.5 Å from D334. Owing to their proximity to the active site and each other we decided to investigate their individual and combined effects, and variants hIDS_A354T, hIDS_H356R, and hIDS_A354T_H356R were included in the study. Moreover, close to equal probabilities were predicted for valine and isoleucine at position 329 in IDS-A1 and IDS-A3, and both variants were included for each ancestor. As probabilities slightly favored valine, those sequences were denoted IDS-A1 and IDS-A3, and the corresponding variants, IDS-A1_V329I and IDS-A3_V329I. An overview of all variants included in the study and their respective number of mutations can be found in Table S1. All enzymes could be successfully expressed in ExpiCHO cells (yield of 30–140 mg/L cell culture) and were purified by affinity chromatography and size exclusion chromatography (Figure S4).

### Two ancestral enzymes display increased activity compared with hIDS and idursulfase

**Figure 3:**
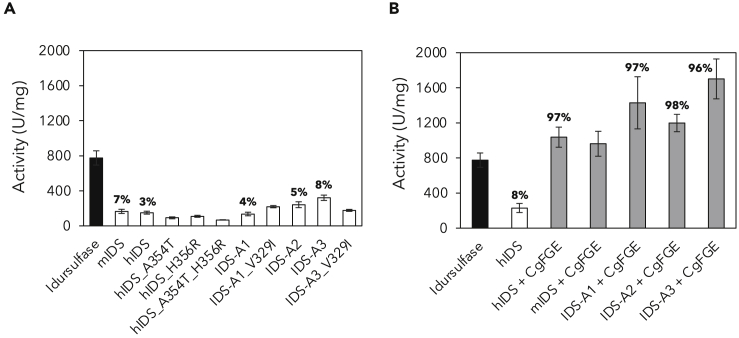
Activity and formylglycine content of IDS enzymes (A) Specific activities of IDS enzymes; error bars show the standard deviations from 3 to 12 replicates. The percentage of formylglycine as determined by mass spectrometry is shown in bold above the bars for the respective variants. (B) Specific activities of IDS enzymes co-expressed with FGE from *C. griseus* (*Cg*FGE). Average activities are shown for two independent transfection experiments for each variant, and error bars show the standard deviations from 6 to 12 replicates. The percentage of formylglycine as determined by mass spectrometry is shown in bold above the bars for the respective variants. See also Figures S1 and S6 and Table S1.

Activity measurements were performed using a coupled assay (Figure S1B) with α-L-iduronidase and artificial substrate 4-methylumbelliferyl-α-L-idopyranosiduronic acid-2-sulfate disodium salt (4-MU-αIdoA2S) (Voznyi et al., 2001). Little difference was observed between hIDS and mIDS, and the hIDS variants displayed slightly lower activities than hIDS (Figure 3A). However, ancestral enzymes (except IDS-A1) showed higher activities than hIDS and mIDS, with an increase of more than 2-fold for IDS-A3. Despite overall low percentages of enzymes with modified Cys84 (expressed as percentage of formylglycine, or %fGly), a correlation could be seen with the specific activity for the ancestral enzymes. This suggested that the higher activities may reflect the percentage of active enzyme in the batches (Figure 3A).

To investigate the significance of increased fGly content in the ancestral enzymes, hIDS, mIDS, and the three original ancestral enzymes were co-expressed with FGE from *Cricetulus griseus* (*Cg*FGE), and an overview of the activity measurements is shown in Figure 3B. Co-expression with *Cg*FGE resulted in a 4.5-fold increase in activity for hIDS, reaching higher activity levels than idursulfase under the same conditions. Interestingly, a similar trend as in the first expression experiment was observed for activity in ancestors when compared with hIDS; activity of IDS-A3 was almost 2-fold higher. Determination of fGly content for some of the enzymes showed that all that were co-expressed with *Cg*FGE had more than 95% fGly (Figure 3B), highlighting the functional and efficient interaction between ancestral enzymes and modern *Cg*FGE. The generally high fGly content in our enzymes might explain their increased activity when compared with idursulfase (for which activity was similar to previously reported values [Demydchuk et al., 2017]), as its %fGly had been determined to be 50% (Muenzer et al., 2006) and 68% (Chung et al., 2014). Overall, no correlation between activity and fGly content was observed in the second expression experiment as formation of fGly was essentially complete.

Thermostability of the enzymes was characterized using nanoDSF (Magnusson et al., 2019; Martin et al., 2014) (Table 1). The melting temperatures were relatively high for all enzymes, and only small differences were found—likely due to the small differences in sequence identities between the enzymes. The data showed that all enzymes with lower *T*_m_ values than hIDS contained mutation A354T, which is one of two mutations close to the active site of hIDS (Figure 2) and was predicted for IDS-A3. A new variant of IDS-A3 with reversed mutation was designed (IDS-A3_T354A), for which the melting temperature was determined to be 74.4°C (Table 1, melting curves can be found in Figure S5). Variant IDS-A3_T354A was expressed in ExpiCHO alongside hIDS and IDS-A3, both with and without human FGE (*Hs*FGE). Activities of IDS-A3 and IDS-A3_T354A expressed without *Hs*FGE were similar, and both were higher compared with hIDS (Figure S6). The IDS-A3 variants expressed with *Hs*FGE were also more active than hIDS expressed with *Hs*FGE; however, IDS-A3 displayed higher activity than IDS-A3_T354A. Overall, activities of hIDS and IDS-A3 co-expressed with *Hs*FGE were similar to those upon co-expression with *Cg*FGE.

**Table 1:** Melting temperatures of all enzymes as determined by nanoDSF

	*T*_m_ (°C)
mIDS	73.3 ± 0.1
hIDS	73.3 ± 0.2
hIDS_A354T	69.8 ± 0.1
hIDS_H356R	74.6 ± 0.1
hIDS_A354T_H356R	71.9 ± 0.1
IDS-A1	73.9 ± 0.1
IDS-A1_V329I	73.9 ± 0.3
IDS-A2	75.1 ± 0.1
IDS-A3	67.8 ± 0.1
IDS-A3_V329I	69.0 ± 0.6
IDS-A3_T354A	74.4 ± 0.5

See also Figure S5.

**Figure 4:**
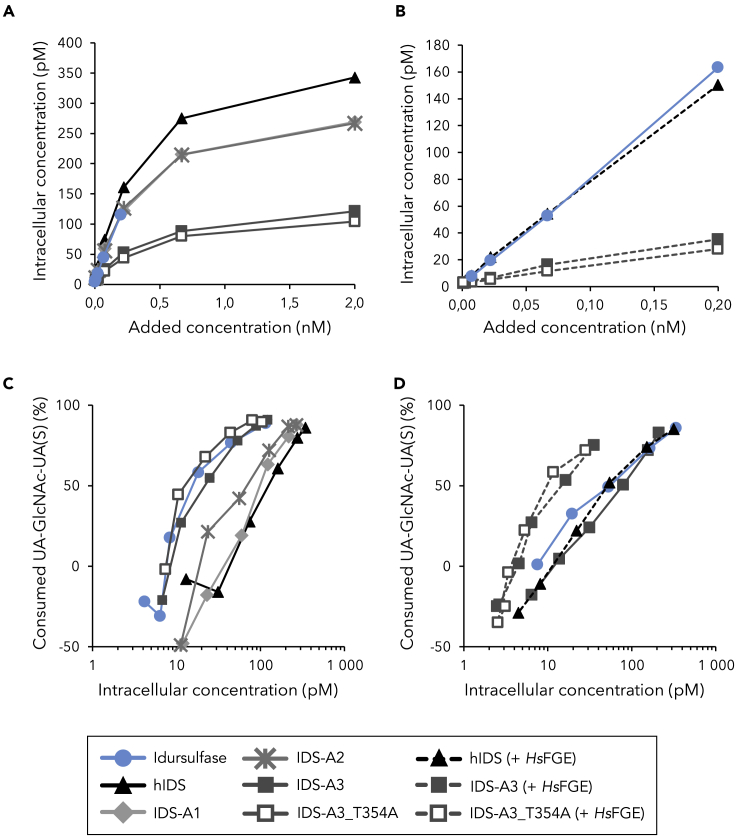
Intracellular concentrations of IDS variants and degradation of heparan sulfate in fibroblasts of patients with MPS II (A) Intracellular concentrations of enzymes expressed without *Hs*FGE 24 h after treatment. The saturation indicates receptor-mediated uptake of all IDS variants. (B) Intracellular concentrations of enzymes expressed with *Hs*FGE 24 h after treatment. Different concentrations were used due to differences in activity of one order of magnitude. (C) Consumed substrate UA-GlcNAc-UA(S) (when compared with internal standard chondroitin disaccharide Δdi-4S sodium) as a function of intracellular enzyme concentration for enzymes expressed without *Hs*FGE. (D) Consumed substrate UA-GlcNAc-UA(S) (when compared with the internal standard) as a function of intracellular enzyme concentration for enzymes expressed with *Hs*FGE. For clarity, the intracellular enzyme concentration is shown on a logarithmic axis in (C and D). Based on biological duplicates the average errors of intracellular concentration and consumed substrate are estimated to be 8% and 33%, respectively. Errors were smaller for high enzyme concentrations and larger for low enzyme concentrations.

Encouraged by the *in vitro* activity data, we aimed to verify the increased activity of ancestral enzymes *ex vivo*. Fibroblasts of patients with MPS II were incubated with IDS variants expressed with and without *Hs*FGE, followed by determination of intracellular enzyme concentration and consumed substrate (Figure 4). Figures 4A and 4B show that intracellular concentrations of IDS-A3 and IDS-A3_T354A after 24 h were substantially lower than those of the other enzymes, including IDS-A2. IDS-A3 and IDS-A3_T354A that were not expressed with *Hs*FGE clear similar amounts of substrate as idursulfase (Figure 4C), despite displaying lower activity in *in vitro* activity assays (Figure 3A). hIDS, IDS-A1 and IDS-A2 have similar intracellular activities as idursulfase and IDS-A3 variants, which is in accordance with *in vitro* activity. All IDS-A3 and IDS-A3_T354A batches that were co-expressed with *Hs*FGE clear more substrate than idursulfase and hIDS + *Hs*FGE (Figure 4D), which is also in accordance with activity measurements. These results highlight that modern *Hs*FGE is capable of catalyzing the modification of the active site cysteine in reconstructed ancestral enzymes, which are functional in a complex intracellular system.

### Glycosylation profiles are conserved in ancestral enzymes

**Figure 5:**
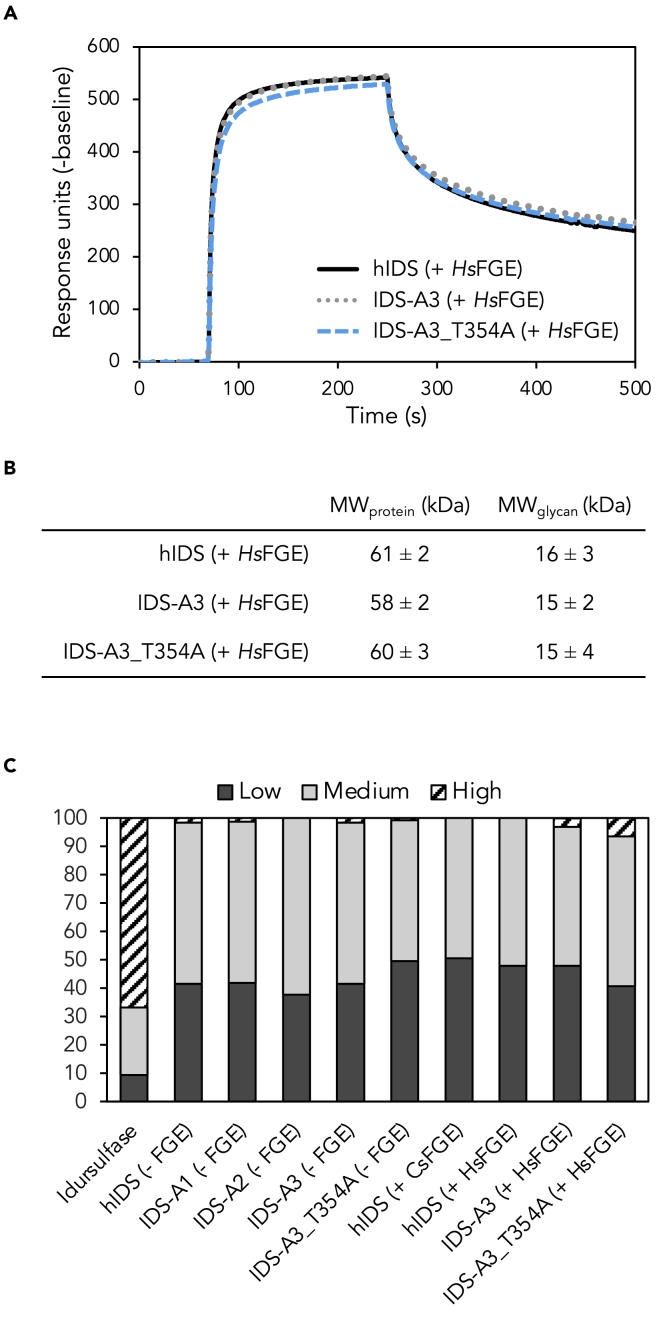
Investigation of glycan profiles (A) Binding of hIDS, IDS-A3 and IDS-A3_T354A co-expressed with *Hs*FGE to the recombinant human M6P receptor was analyzed by surface plasmon resonance. Measurements were performed on a Biacore T200 system using the single cycle kinetics mode. *K*_D_ values were determined to be 32 nM (hIDS), 26 nM (IDS-A3), and 46 nM (IDS-A3_T354A). (B) Molecular weights of protein and glycan fractions as determined by SEC-MALS. (C) Profiles of released N-glycans as determined by GlycoWorks RapiFluor-MS N-Glycan Kit. Glycans were divided into three categories based on retention time in LC-FLR-MS: low, medium, and high complexity (relative abundancy shown). The low-complexity N-glycans elute earlier than more complex glycans. Fluorescence traces can be found in Figure S7.

With respect to the decreased intracellular concentrations observed for IDS-A3 and IDS-A3_T354A, we hypothesized that general protein shape or surface properties of the ancestral enzymes could be influenced either directly through mutations or indirectly through alterations to glycosylation caused by ancestral mutations. A series of experiments was conducted to investigate the latter (Figure 5). Figure 5A shows that hIDS, IDS-A3 and IDS-A3_T354A bind to the mannose-6-phosphate receptor (M6PR) with similar association and dissociation patterns, showing that the receptor interaction is functional and therefore likely not the reason for decreased intracellular concentrations of the ancestral enzymes. Moreover, *K*_D_ values for all three enzymes were within one order of magnitude from previously published values (Sonoda et al., 2018). A more detailed investigation of the glycan patterns was performed by size-exclusion chromatography coupled to a multiple angle light scattering detector (SEC-MALS) and *Rapi*Fluor Kit. SEC-MALS analysis showed that the molecular weights of the protein fractions of hIDS, IDS-A3 and IDS-A3_T354A were similar to previously published values for deglycosylated IDS (Wilson et al., 1990) and no significant differences in total glycan weight were found among the three variants (Figure 5B). Analysis of released N-glycans using the *Rapi*Fluor Kit showed that the IDS enzymes presented similar glycan profiles (Figure 5C, fluorescence traces can be found in Figure S7). To facilitate the comparison, glycans were attributed to one of three categories based on their retention times in liquid chromatography with a fluorescence detector coupled to mass spectrometry (LC-FLR-MS): low complexity (eluting before and including mannose-5), medium complexity (eluting after mannose-5 but before sialic acid-containing glycans), and high complexity (glycans with sialic acid). The IDS enzyme profiles were mainly dominated by low- and medium-complexity glycans, as opposed to idursulfase that contained mostly high-complexity glycans. Overall, glycan profiles and M6PR binding characteristics were highly similar among all IDS variants and did not provide any indications as to what might cause the decreased intracellular concentrations observed for IDS-A3 and IDS-A3_T354A.

**Figure 6:**
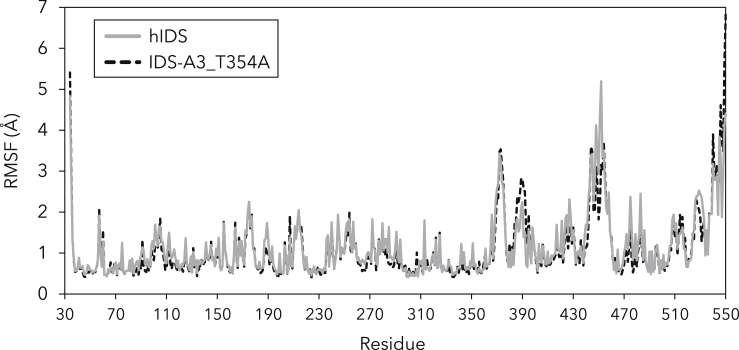
Molecular dynamics simulations of hIDS and IDS-A3_T354A Root-mean-square fluctuation (RMSF) of hIDS and IDS-A3_T354A during molecular dynamics (MD) simulations. The RMSF values are the average of three independent 100-ns MD trajectories for each enzyme.

To investigate other possible effects of the ancestral mutations, molecular dynamics (MD) simulations were performed with hIDS and a homology model of IDS-A3_T354A (Figure 6). Overall, the root-mean-square fluctuations (RMSF) per residue over 100-ns MD trajectories are highly similar for both enzymes and differences in dynamics only seem to occur between residues 387–392 and residues 448–454. Both stretches are situated in flexible loops on the enzyme surface, the second loop being the unresolved loop in the crystal structure (PDB: 5FQL) that is susceptible to proteolytic cleavage and separates the two subdomains of IDS (Demydchuk et al., 2017). Both stretches contain ancestral mutations: Q389E and Y452H and P454H, respectively. It should be noted that the starting conformation for the unresolved loop is predicted, resulting in an inherent uncertainty, but the same conformation is used for both hIDS and IDS-A3-T354A. Moreover, the loop is likely to be highly flexible, which may be reflected in the RMSF profile.

## Discussion

Encouraged by evolution-inspired redesign of sulfatases through combination of human and mouse sequences (Simonis et al., 2019), we set out to explore the IDS sequence space between primates and rodents by means of ASR. We hypothesized that ASR could be a way to increase the activity or stability of lysosomal enzymes and thereby enhance their potential for enzyme replacement therapy, as we had previously obtained interesting enzyme scaffold using this method (Hendrikse et al., 2018, 2020). Three putative ancestors and several variants were designed and compared with extant IDS enzymes from *Homo sapiens* and *Mus musculus*. Apart from little difference in stability, we found two ancestral variants that displayed higher activity than hIDS and idursulfase, both in *in vitro* assays as well as in *ex vivo* experiments using patient fibroblasts.

The phylogenetic tree that was used for ancestral reconstruction is mostly in accordance with a species tree that was created with the same set of species (Upham et al., 2019), apart from small differences in the clades wherein the ancestral nodes were chosen—in particular at divergence points that are not resolved in the IDS tree. These unresolved nodes are likely due to a too high degree of conservation between the extant sequences, which is also reflected in the low number of mutations in the ancestors (20 for IDS-A3). The high sequence similarity between the ancestors and the modern human enzyme could be beneficial in a therapeutic setting, where immunogenicity is a common challenge. This advantage had previously been described for ancestral uricases (Kratzer et al., 2014). None of the 20 predicted ancestral mutations occur in any of the 128 positions in the 550-residue protein that harbor reported disease-causing mutations (Demydchuk et al., 2017). We take this as a sign of the robustness of the reconstruction process, as important sites are more likely to be conserved throughout evolution and are also more prone to disease-causing mutations. Analysis of evolutionary conservation throughout the IDS alignment with ConSurf (Armon et al., 2001) shows that most ancestral mutations occur in highly variable positions (Figure S8), which could be expected. However, mutations T146S (close to glycosylation site N144), H356R (that appears to be stabilizing), I360M, and S470A occur in relatively conserved positions. Despite its destabilizing effect, mutation A354T occurs at a site that is highly variable in the alignment.

Based on the initial activity measurements and determination of %fGly (Figure 3A) we hypothesized that the ancestral IDS enzymes may be more prone to modification by FGE. The FGE binds to newly synthesized, unfolded sulfatases in the endoplasmic reticulum and catalyzes the generation of fGly from a cysteine residue through a multistep redox process (Roeser et al., 2006). The sequence motif that is required for FGE binding (C-[TSAC]-PSR) is conserved in all human sulfatases, suggesting a general binding mechanism, but conversion of the cysteine to fGly in the different sulfatases depends on the flanking sequences of approximately 10 residues on either side (Preusser-Kunze et al., 2005). However, no ancestral mutations occur in the vicinity of cysteine 84, and it is thus not obvious that binding to the FGE is altered in the ancestral enzymes. This was supported by activity and %fGly data upon co-expression with *Cs*FGE (Figure 3B), and we concluded that the observed increased activity of especially IDS-A3 when compared with hIDS in the *in vitro* assays was likely due to an increase in catalytic rate. Overall the activity data show that ancestral and modern IDS from primates and rodents are successfully modified by modern FGE from primates and rodents as well.

Increased clearance of substrate by IDS-A3 and its variant IDS-A3_T354A when compared with hIDS and idursulfase was also observed in fibroblasts of patients with MPS II (Figure 4). Both IDS-A3 variants that were not co-expressed with *Hs*FGE cleared similar percentages of substrate as idursulfase in patient fibroblasts, despite lower activity in the *in vitro* assay (Figure S6). The same variants but co-expressed with *Hs*FGE consume more substrate compared with idursulfase and hIDS + *Hs*FGE, which is mostly in accordance with *in vitro* activity measurements. It should be noted that the observed increased clearance of substrate after 24 h could be due to several factors, such as increased catalytic rate, and also increased stability of the enzymes in the lysosome. The experiments in patient fibroblast also revealed a decreased intracellular concentration for IDS-A3 and IDS-A3_T354A when compared with all other enzymes. This could be due to a decreased cellular uptake, which is receptor-mediated through interaction of the M6PR with the glycans on the surface of IDS. Studies have shown that all putative glycosylation sites in IDS are used, but none of them is crucial for activity (Millat et al., 1997). Even though none of the glycosylation sites was found to be essential for cellular uptake and lysosomal targeting of the enzyme, N280 appeared to be the most important site for this purpose. All eight glycosylation sites were conserved in the ancestral IDS enzymes, apart from T146S in the motif around N144 for IDS-A2 and IDS-A3. Moreover, no mutations occur in the direct vicinity of N280, making it unlikely that ancestral mutations disturb the overall glycosylation in ancestral IDS, as supported by the data in Figure 5. No sialic acid-containing glycans were detected in any of the IDS samples that had been produced in ExpiCHO (Figure S7), which we had previously experienced with expression of other lysosomal enzymes in this strain (unpublished data). It has been posed that sialylation is important for antibody recognition/immunogenicity and circulating half-life of IDS (Chung et al., 2014).

In case that cellular uptake is influenced directly by ancestral mutations on the surface, the difference between IDS-A2 and IDS-A3 is of particular interest. Only 10 positions differ between the two sequences, of which 7 are located on the surface of the enzyme: L189V(IDS-A2)/A(IDS-A3), E254Q, S369P, S397T, P454H, I472F(IDS-A2)/S(IDS-A3), and F545P (and likely Y452H, which is located in the unresolved loop in the structure [Demydchuk et al., 2017]). Changes in surface charge occur due to E254Q and possibly P454H and Y452H, which are located in the unresolved loop in the structure (PDB: 5FQL). E254 is spatially close to flexible region 270–280, which includes glycosylated residue N280 that is hypothesized to be of importance for cellular uptake. The decreased intracellular concentration of IDS-A3 variants could be a possible advantage as it implies a prolonged systemic circulation, or plasma half-life, for these enzymes. Studies with other sulfatases have shown that prolonged systemic circulation may result in increased brain uptake (Gustavsson et al., 2019). The obstacle of treating CNS manifestations of MPS II remains one of the biggest challenges to be met at the present time, and various approaches to improve distribution are actively investigated, such as the use of other receptors and endocytic mechanisms (Chen et al., 2019; Sonoda et al., 2018).

We have shown that ASR can successfully be applied to enzymes in complex systems that function in concert with extant enzymes and receptors in cells. Two ancestral lysosomal enzymes were found to have increased activity *in vitro* and *ex vivo* when compared with idursulfase, the enzyme that is currently available for ERT. This increase in activity would have been difficult to achieve by other protein engineering strategies, as the identification and selection of positions to mutate as well as the resulting ancestral residues is not an obvious process. Most importantly, the activity increase allows for a decreased dose and shorter intravenous administration times in ERT, which are both major advantages for potential therapeutic enzymes. We believe these results are highly encouraging as they demonstrate the potential of ASR for improving therapeutic properties of lysosomal enzymes for ERT, a therapeutic area with a significant unmet medical need.

### Limitations of the study

Ancestral sequence reconstruction is a relatively young bioinformatics method: updated methods, models, and software are continuously developed. Owing to the time that is needed to carry out the experimental work to verify the computational results, some of the methods or programs may have been further developed. We acknowledge that even though our model searches in two different software programs suggested a gamma distribution as optimal for our data, there may be advanced models that more accurately describe among-site rate heterogeneity in our dataset.

### Resource availability

#### Lead contact

Further information and requests for resources and reagents should be directed to and will be fulfilled by the Lead Contact, Erik Nordling (Erik.Nordling@sobi.com).

#### Materials availability

All protein sequences can be found in the Supplemental Data Items and the corresponding proteins can be obtained as described in the Transparent methods section.

#### Data and code availability

The published article includes all datasets (sequences) generated and analyzed during this study.

## Methods

All methods can be found in the accompanying Transparent methods supplemental file.

## Supplemental information

Document S1. Transparent methods and Figures S1–S8
