# Supplementary material for: Ancestral lysosomal enzymes with increased activity harbor therapeutic potential for treatment of Hunter syndrome
Source: iScience. 2021 Feb 6;24(3):102154. doi: 10.1016/j.isci.2021.102154 (PMC7907806; doi:10.1016/j.isci.2021.102154)
Supplement: Document S1. Transparent methods and Figures S1–S8 [file mmc1.pdf]

iScience, Volume *24*

Supplemental information

Ancestral lysosomal enzymes with increased activity harbor therapeutic potential for treatment of Hunter syndrome

Natalie M. Hendrikse, Anna Sandegren, Tommy Andersson, Jenny Blomqvist, Åsa Makower, Dominik Possner, Chao Su, Niklas Thalén, Agneta Tjernberg, Ulrica Westermark, Johan Rockberg, Stefan Svensson Gelius, Per-Olof Syrén, and Erik Nordling

Supplemental Data Items

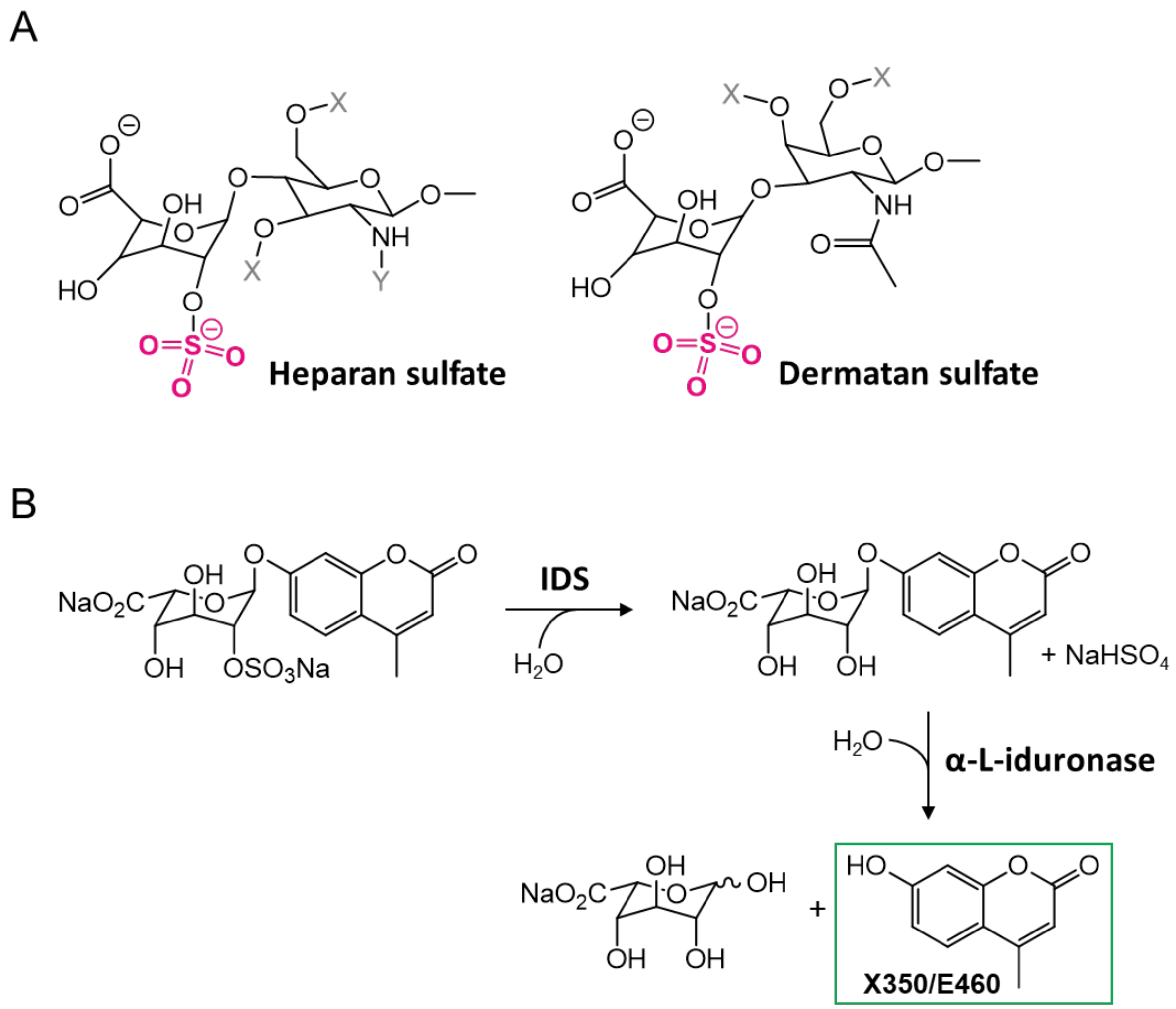

Figure S1: Natural and artificial substrates of IDS, related to Figure 3. A) Natural substrates heparan sulfate (**α**1-4 linkage) and dermatan sulfate (**α**1-3 linkage). The sulfate group that is removed by IDS is indicated in pink. Variable substituents are indicated by X and Y, where X can be either H or SO_3^- and Y can be H, SO_3^- or $COCH_3$. **B)** Artificial substrate 4-methylumbelliferyl-**α**-L-idopyranosiduronic acid-2-sulphate disodium salt (4-MU-**α**IdoA2S) is converted to 4-MU- **α**-iduronide by IDS, followed by hydrolysis by **α**-L-iduronidase to release 4-methylumbelliferone. 4-methylumbelliferone (4-MU) can be detected in a fluorometric assay through excitation at 350 nm and measuring emission at 460 nm.

A

```
                                1                                                 50
Homo_sapiens                    ------MPPPRTGRGLLWLGLVLSSVCVALGSETQANSTT----------
Pan_troglodytes                 ------MPPPRTGRGLPWLGLVLSSVCVALGSETQANSTT----------
Gorilla_gorilla_gorilla         --------------------------------------------------
Nomascus_leucogenys             ------MSPPRTGQGLLWLGVVLSSVCVAXVTSPKPPSFV----------
Pongo_abelii                    ------MPPPRTGRGLLWLGLVLSSVCVALGSETQADSTT----------
Macaca_mulatta                  ------MPTPGSGRGFLWLGLVLSSVCVALGCETQANSTT----------
Macaca_fascicularis             ------MPTPGSGRGFLWLGLVLSSVCVALGCETQANSTT----------
Papio_anubis                    ------MPTPGSGRGFLWLGLVLSSVCVALGCEMQANSTT----------
Chlorocebus_sabaeus             ------MPTPGSGRGFLWLGLVLSSVCVALGSETQANSTT----------
Callithrix_jacchus              ------MPPPRPSRCLLLLGLVLGSVCVTLGSQAQASSTT----------
Neotoma_lepida                  MSL--PPPPPQPGWRLLGFSLLLGSFCFALESVALGNSAT----------
Mesocricetus_auratus            MSQMPPPPPPLLGWRLLGFSLLLSSFCFSLESAALDNSST----------
Rattus_norvegicus               -------------WRQLSFSLLLGFFCIALVSAAQGNSAT----------
Mus_musculus                    --M--SPPPPPPIWRQLSFSLLLGSFCIALESAAQGNSAT----------
Dipodomys_ordii                 ------MPLTPPAWLLLWLSLLLSSVCPALKTAAQASAST----------
Fukomys_damarensis              -------MLLPPGWPLLWLGLVLGSSCANLESAAQVNSTTGLKSWPNPKS
Heterocephalus_glaber           -------MLPPPAWPLLWLGLVLGSCCADLEAAAQVNSST----------
Cavia_porcellus                 ---------PPPGWPLLWLGLLLSS-SAVLESAAQVNSAT----------
Bos_taurus                      --------MPPPSRSLLWLGLVLGAVCASRGPAAPGN-AT----------
Bos_mutus                       --------------------------------------------------
Cervus_elaphus_hippelaphus      --------MPPPSRSLLWLGLVLGAVCASREPAAPGN-AT----------
Desmodus_rotundus               --------MPLLDRSLLWLSLVLGS-CASLGSAARGNNAK----------
Myotis_lucifugus                --V--SPPGPAPSGARLATGLVLGSFCTLLGSVAPGNNSK----------
Myotis_brandtii                 --------MPLSDRGLLLLGLVLGSFCTLLGSVAPGNNSK----------
Myotis_davidii                  --------MPLSDRGLLLLGLVLGSFCTLLGSVVPGNNSK----------
Pteropus_alecto                 --------MLPPGWGLLWLGLVLGSTYVSLGSCNLASNSK----------
Felis_catus                     ---------PPAGQCLLWLGLVLASVCASLESAAPSNSTT----------
Ailuropoda_melanoleuca          --A--GVEMPPAGWCLLWLGLVLASVCASLESAAPSNSTT----------
Canis_lupus_familiaris          --------MPPGGWCLLCFGLVLSSVCASAESAAPSNLTT----------
Neovison_vison                  ------------------LGLVLASVCTSLESAASSNSTT---------

                                51                                               100
Homo_sapiens                    ------------------------------DALNVLLIIVDDLRPSLGCY
Pan_troglodytes                 ------------------------------DALNVLLIIVDDLRPSLGCY
Gorilla_gorilla_gorilla         --------------------------------------------------
Nomascus_leucogenys             ------------------------------DALNVLLIIVDDLRPSLGCY
Pongo_abelii                    ------------------------------DGLNVLLIIVDDLRPSLGCY
Macaca_mulatta                  ------------------------------DALNILLIIVDDLRPSLGCY
Macaca_fascicularis             ------------------------------DALNILLIIVDDLRPSLGCY
Papio_anubis                    ------------------------------DALNILLIIVDDLRPSLGCY
Chlorocebus_sabaeus             ------------------------------DALNILLIIVDDLRPSLGCY
Callithrix_jacchus              ------------------------------DALNVLLIIVDDLRPSLGCY
Neotoma_lepida                  ------------------------------DALNILLIIVDDLRPSLGCY
Mesocricetus_auratus            ------------------------------DALNVLLIIVDDLRPSLGCY
Rattus_norvegicus               ------------------------------DALNILLIIVDDLRPSLGCY
Mus_musculus                    ------------------------------DALNILLIIVDDLRPSLGCY
Dipodomys_ordii                 ------------------------------GALNVLLIIVDDLRPSLGCY
Fukomys_damarensis              ATHTSGFLCILAPSVVTHIPGGFPLWLGCQDAPNVLLIIVDDLRPSLGCY
Heterocephalus_glaber           ------------------------------DARNVLLIIVDDLRPSLGCY
Cavia_porcellus                 ------------------------------DALNVLLIIVDDLRPSLGCY
Bos_taurus                      ------------------------------DPLNVLLIIVDDLRPSLGCY
Bos_mutus                       ------------------------------DPLNVLLIIVDDLRPSLGCY
Cervus_elaphus_hippelaphus      ------------------------------DPLNVLLIIVDDLRPSLGCY
Desmodus_rotundus               ------------------------------DALNVLLIIVDDLRLSLGCY
Myotis_lucifugus                ------------------------------DALNVLLIIVDDLRLSLGCY
Myotis_brandtii                 ------------------------------DALNVLLIIVDDLRLSLGCY
Myotis_davidii                  ------------------------------DALNVLLIIVDDLRLSLGCY
Pteropus_alecto                 ------------------------------DALNVLLIIVDDLRPSLGCY
Felis_catus                     ------------------------------GPLNVLLIIVDDLRPSLGCY
Ailuropoda_melanoleuca          ------------------------------APLNVLLIIVDDLRPSLGCY
Canis_lupus_familiaris          ------------------------------APLNVLLIIVDDLRPSLGCY
Neovison_vison                  ------------------------------APLNVLLIIVDDLRPSLGCY
```

```
                                 101                                              150
Homo_sapiens                     GDKLVRSPNIDQLASHSLLFQNAFAQQAVCAPSRVSFLTGRRPDTTRLYD
Pan_troglodytes                  GDKLVRSPNIDQLASHSLLFQNAFAQQAVCAPSRVSFLTGRRPDTTRLYD
Gorilla_gorilla_gorilla          -DELVKAPNIDQLASHSLLFQNAFAQQAVCAPSRVSFLTGRRPDTTRLYD
Nomascus_leucogenys              GDKLVRSPNIDQLASHSLLFQNAFAQQAVCAPSRVSFLTGRRPDTTRLYD
Pongo_abelii                     GDKLVRSPNIDQLASHSLLFQNAFAQQAVCAPSRVSFLTGRRPDTTRLYD
Macaca_mulatta                   GDKLVRSPNIDQLASHSLLFQNAFAQQAVCAPSRVSFLTGRRPDTTRLYD
Macaca_fascicularis              GDKLVRSPNIDQLASHSLLFQNAFAQQAVCAPSRVSFLTGRRPDTTRLYD
Papio_anubis                     GDKLVRSPNIDQLASHSLLFQNAFAQQAVCAPSRVSFLTGRRPDTTRLYD
Chlorocebus_sabaeus              GDKLVRSPNIDQLASHSLLFQNAFAQQAVCAPSRVSFLTGRRPDTTRLHN
Callithrix_jacchus               GDKLVRSPNIDQLASHSLLFQNAFAQQAVCAPSRVSFLTGRRPDTTRLYD
Neotoma_lepida                   GDKLVRSPNIDQLASHSILFQNAFAQQAVCAPSRVSFLTGRRPDTTRLYD
Mesocricetus_auratus             GEKVVRSPNIDQLASHSVLFQNAFAQQAVCAPSRVSFLTGRRPDTTRLYD
Rattus_norvegicus                GDKLVRSPNIDQLASHSIVFENAFAQQAVCAPSRVSFLTGRRPDTTRLYD
Mus_musculus                     GDKLVRSPNIDQLASHSVLFQNAFAQQAVCAPSRVSFLTGRRPDTTRLYD
Dipodomys_ordii                  GDKIVRSPNIDQLAAHSLLFQNAFAQQAVCAPSRVSFLTGRRPDTTRLYD
Fukomys_damarensis               GDKAVKSPNIDQLASHSLLFQNAFAQQAVCAPSRVSFLTGRRPDTTRLYD
Heterocephalus_glaber            GDKLVKSPNIDQLASHSLLFQNAFAQQAVCAPSRVSFLTGRRPDTTRLYD
Cavia_porcellus                  GDKLVKSPNIDQLASHSLLFQNAFAQQAVCAPSRVSFLTGRRPDTTRLYD
Bos_taurus                       GNKLIRSPNIDQLASRSLLFQNAFAQQAVCAPSRVSFLTGRRPDTTRLYD
Bos_mutus                        GNKLIRSPNIDQLASRSLLFQNAFAQQAVCAPSRVSFLTGRRPDTTRLYD
Cervus_elaphus_hippelaphus       GNKLIRSPNIDQLASRSLLFQNAFAQQAVCAPSRVSFLTGRRPDTTRLYD
Desmodus_rotundus                GDKLVKSPNIDQLASHSLLFQNAFAQQAVCAPSRVSFLTGRRPDTTRLYD
Myotis_lucifugus                 GDKLIRSPNIDQLASHSLLFQNAFAQQAVCAPSRVSFLTGRRPDTTRLYD
Myotis_brandtii                  GDKLIRSPNIDQLASHSLLFQNAFAQQAVCAPSRVSFLTGRRPDTTRLYD
Myotis_davidii                   GDKLIRSPNIDQLASHSLLFQNAFAQQAVCAPSRVSFLTGRRPDTTRLYD
Pteropus_alecto                  GDKLIRSPNIDQLASHSLLFWNAFAQQAVCAPSRVSFLTGRRPDTTRLYD
Felis_catus                      GDKAIRSPNIDQLASRSLLFQNAFAQQAVCAPSRVSFLTGRRPDTTRLYD
Ailuropoda_melanoleuca           GDKLIRSPNIDQLASHSLLFQNAFAQQAVCAPSRVSFLTGRRPDTTRLYD
Canis_lupus_familiaris           GDKLVRSPNIDQLASHSLLFQNAFAQQAVCAPSRVSFLTGRRPDTTRLYD
Neovison_vison                   GDKLVRSPNIDQLASHSLLFQNAFAQQAVCAPSRVSFLPGRRPDTTRLYD
                                  :: :::*******::*::* *****************.*********::

                                 151                                              200
Homo_sapiens                     FNSYWRVHAGNFSTIPQYFKENGYVTMSVGKVFHP-GISSNHTDDSPYSW
Pan_troglodytes                  FNSYWRVHAGNFSTIPQYFKENGYVTMSVGKVFHP-GISSNHTDDSPYSW
Gorilla_gorilla_gorilla          FNSYWRVHAGNFSTIPQYFKENGYVTMSVGKVFHPGGISSNHTDDSPYSW
Nomascus_leucogenys              FNSYWRVHAGNFSTIPQYFKENGYVTMSVGKVFHP-GISSNHTDDSPYSW
Pongo_abelii                     FNSYWRVHAGNFSTIPQYFKENGYVTMSVGKVFHP-GISSNHTDDSPYSW
Macaca_mulatta                   FNSYWRVHAGNFSTIPQYFKENGYVTMSVGKVFHP-GITSNHTDDSPYSW
Macaca_fascicularis              FNSYWRVHAGNFSTIPQYFKENGYVTMSVGKVFHP-GITSNHTDDSPYSW
Papio_anubis                     FNSYWRVHAGNFSTIPQYFKENGYVTMSVGKVFHPEGITSNHTDDSPYSW
Chlorocebus_sabaeus              FNSYWRVHAGNFSTIPQYFKENGYVTMSVGKVFHP-GITSNHTDDSPYSW
Callithrix_jacchus               FNSYWRVHAGNFSTIPQYFKDNGYVTMSVGKVFHP-GISSNHSDDSPYSW
Neotoma_lepida                   FNSYWRVHSGNFSTIPQYFKENGYVTMSVGKVFHP-GISSNHSDDSPYSW
Mesocricetus_auratus             FNSYWRVHSGNFSTIPQYFKENGYVTMSVGKVFHP-GISSNHSDDSPYSW
Rattus_norvegicus                FNSYWRVHSGNFSTIPQYFKENGYVTMSVGKVFHP-GISSNHSDDYPYSW
Mus_musculus                     FNSYWRVHSGNFSTIPQYFKENGYVTMSVGKVFHP-GISSNHSDDYPYSW
Dipodomys_ordii                  FNSYWRSHSGNFSTIPQYFKENGYVTMSVGKVFHP-GISSNYSDDSPYSW
Fukomys_damarensis               FNSYWRVHSGNFSTLPQYFKENGYVTLSVGKVFHP-GISSNHTDDSPYSW
Heterocephalus_glaber            FNSYWRVHSGNFSTLPQYFKENGYVTLSVGKVFHP-GISSNHSDDSPYSW
Cavia_porcellus                  FGSYWRVHAGNFSTLPQHFKENGYVTMSVGKVFHP-GISSNHSDDSPYSW
Bos_taurus                       FNSYWRVHAGNFSTIPQYFKENGYVTMSVGKVFHP-GISSNHSDDSPYSW
Bos_mutus                        FNSYWRVHAGNFSTIPQYFKENGYVTMSVGKVFHP-GISSNHSDDSPYSW
Cervus_elaphus_hippelaphus       FNSYWRVHAGNFSTIPQYFKENGYVTMSVGKVFHP-GISSNHSDDSPYSW
Desmodus_rotundus                FNSYWRVQAGNFSTIPQYFKENGYVTMSVGKVFHP-GISSNHSDDSPYSW
Myotis_lucifugus                 FNSYWRVQAGNFSTIPQYFKENGYVTMSVGKVFHP-GISSNHSDDSPYSW
Myotis_brandtii                  FNSYWRVQAGNFSTIPQYFKENGYVTMSVGKVFHP-GISSNHSDDFPYSW
Myotis_davidii                   FNSYWRVQAGNFSTIPQYFKENGYMTMSVGKVFHP-GISSNHSDDSPYSW
Pteropus_alecto                  FNSYWRAHAGNFSTIPQYFKENGYVTMSVGKVFHP-GISSNHSDDSPYSW
Felis_catus                      FNSYWRIHAGNFSTIPQYFKENGYVTMSVGKVFHP-GISSNYSDDSPYSW
Ailuropoda_melanoleuca           FNSYWRVHAGNFSTIPQYFKENGYVTMSVGKVFHP-GISSNYSDDSPYSW
Canis_lupus_familiaris           FNSYWRVHAGNFSTLPQYFKENGYVTMSVGKVFHP-GISSNYSDDSPYSW
Neovison_vison                   FNSYWRVHAGNFSTIPQHFKENGYVTMSVGKVFHP-GISSNYSDDSPFSW
                                 *.**** ::*****:**:**:***:*:******** **:**::** *:**
```

```
                             201                                              250
Homo_sapiens                 SFPPYHPSSEKYENTKTCRGPDGELHANLLCPVDVLDVPEGTLPDKQSTE
Pan_troglodytes              SFPPYHPSSEKYENTKTCRGPDGELHANLLCPVDVLDVPEGTLPDKQSTE
Gorilla_gorilla_gorilla      SFPPYHPSSEKYENTKTCRGPDGELHANLLCPVDVLDVPEGTLPDKQSTE
Nomascus_leucogenys          SFPPYHPSSXXXXXXKTCRGPDGELHANLLCPVDVLDVPEGTLPDKQSTE
Pongo_abelii                 SFPPYHPSSEKYENTKTCRGPDGELHANLIAKKMCWMFPRAPCCDKQSTE
Macaca_mulatta               SFPPYHPSSEKYENTKTCRGPDGELHANLLCPVDVVDVPEGTLPDKQSTE
Macaca_fascicularis          SFPPYHPSSEKYENTKTCRGPDGELHANLLCPVDVVDVPEGTLPDKQSTE
Papio_anubis                 SFPPYHPSSEKYENTKTCRGPDGELHANLLCPVDVVDVPEGTLPDKQSTE
Chlorocebus_sabaeus          SFPPYHPSSEKYENTKTCRGPDGELHANLLCPVDVVDVPEGTLPDKQSTE
Callithrix_jacchus           SFPPYHPSSEKYENTKTCRGPDGELHANLLCPVDVVDVPEGTLPDKQSTE
Neotoma_lepida               SFPPYHPSTEKYENTKTCRGQDGKLHANLLCPVDVADVPEGTLPDKQSTE
Mesocricetus_auratus         SFPPYHPSSEKYENTKTCRGQDGKLHANLLCPVDVADVPEGTLPDKQSTE
Rattus_norvegicus            SFPPYHPSSEKYENTKTCKGQDGKLHTNLLCPVDVADVPEGTLPDKQSTE
Mus_musculus                 SFPPYHPSSEKYENTKTCKGQDGKLHANLLCPVDVADVPEGTLPDKQSTE
Dipodomys_ordii              SFLPYHPSSEKYENTKTCRGQDGELHANLLCPVDVKDVPEGTLPDIQSTE
Fukomys_damarensis           SFPPYHPSSEKYENTKTCRGQDGELHANLICPVDMADVPEGTLPDKQSTE
Heterocephalus_glaber        SFPPYHPSSEKYENTKTCRGQDGELHANLICPVDMADVPEGTLPDKQSTE
Cavia_porcellus              SLPPYHPSSEKYENTKTCRGQDGELHANLICPVDMADVPEGTLPDKQSTE
Bos_taurus                   SVPPYHPSSEKYENTKTCRGPDGELHANLLCPVDVVDVPEGTLPDKQSTE
Bos_mutus                    SVPPYHPSSEKYENTKTCRGPDGELHANLLCPVDVVDVPEGTLPDKQSTE
Cervus_elaphus_hippelaphus   SVPPYHPSSEKYENTKTCRGPDGELHANLLCPVDVVDVPEGTLPDMQSTE
Desmodus_rotundus            SVPPYHPSSEKYENTKTCRGPDGELHANLICPVDMADIPEGTLPDKQSTK
Myotis_lucifugus             SVLPYHPSSEKYENTKTCKGPDGELHANLICPVDMADIPEGTLPDKQSTK
Myotis_brandtii              SVLPYHPSSEKYENTKTCKGPDGELHANLICPVDMADIPEGTLPDKQSTK
Myotis_davidii               SVPPYHPSSEKYENTKTCKGPDGELHANLICPVDMADIPEGTLPDKQSTK
Pteropus_alecto              SIPPYHPSSEKYENTKTCRGPDGELHANLLCPVDVADVPEGTLPDKQSTE
Felis_catus                  SFPPYHPSSEKYENTKTCRGPDGQLHANLLCPVDVADVPEGTLPDKQSTE
Ailuropoda_melanoleuca       SIPPYHPSSEKYENTKTCRGPDGELHANLLCPVDVADVPEGTLPDKQSTE
Canis_lupus_familiaris       SIPPYHPSSEKYENTKTCRGPDGELHANLLCPVDIADVPEGTLPDKQSTE
Neovison_vison               SVPPYHPSSEKYENTKTCRGPDGELHANLLCPVDVADVPEGTLPDKQSTE
                             *. *****:      ***:* **:**:**:.      .*...  * ***:

                             251                                              300
Homo_sapiens                 QAIQLLEKMKTSASPFFLAVGYHKPHIPFRYPKEFQKLYPLENITLAPDP
Pan_troglodytes              QAIQLLEKMKTSASPFFLAVGYHKPHIPFRYPKEFQKLYPLENITLAPDP
Gorilla_gorilla_gorilla      QAIQLLEKMKTSASPFFLAVGYHKPHIPFRYPKEFQKLYPLENITLAPDP
Nomascus_leucogenys          QAIQLLEKMKTSASPFFLAVGYHKPHIPFRYPKEFQKLYPLENITLAPDP
Pongo_abelii                 QAIQLLEKMKTSASPFFLAVGYHKPHIPFRYPKEFQKLYPLENITLAPDP
Macaca_mulatta               QAIQLLEKMKTSASPFFLAVGYHKPHIPFRYPKEFQKLYPLENITLAPDS
Macaca_fascicularis          QAIQLLEKMKTSASPFFLAVGYHKPHIPFRYPKEFQKLYPLENITLAPDS
Papio_anubis                 QAIQLLEKMKTSASPFFLAVGYHKPHIPFRYPKEFQKLYPLENITLAPDS
Chlorocebus_sabaeus          QAIQLLEKMKTSASPFFLAVGYHKPHIPFRYPKEFQKLYPLENITLAPDS
Callithrix_jacchus           EAIRLLEKMKTSASPFFLAVGYHKPHIPFRYPKEFQKLYPLENITLAPDP
Neotoma_lepida               EAIRLLEKMKTSSSPFFLAVGYHKPHIPFRYPKEFQKLYPLENITLAPDP
Mesocricetus_auratus         EAIRLLEKMKTSASPFFLVVGYHKPHIPFRYPKEFQKLYPLENITLAPDP
Rattus_norvegicus            EAIRLLEKMKTSVSPFFLAVGYHKPHIPFRYPKEFQKLYPLENMTLAPDP
Mus_musculus                 EAIRLLEKMKTSASPFFLAVGYHKPHIPFRYPKEFQKLYPLENITLAPDP
Dipodomys_ordii              EAIRLLEKMKTLSSPFFLAIGYHKPHIPFRYPKEFQKLYPLENITLAPDP
Fukomys_damarensis           EAIRLLEKMKTSASPFFLAVGYHKPHIPFRYPKEFQKLYPLENITLAPDP
Heterocephalus_glaber        EAIRLLEKMKTSASPFFLAVGYHKPHIPFRYPKEFQKLYPLENITLAPDP
Cavia_porcellus              EAICLLEKMKTSASPFFLAVGYHKPHIPFRYPKEFQKLYPLENITLAPDP
Bos_taurus                   QAIQLLGKMKTSASPFFLAVGYHKPHIPFRYPKEFQKLYPLENVSLAPDP
Bos_mutus                    QAIQLLGKMKTSASPFFLAVGYHKPHIPFRYPKEFQKLYPLENVSLAPDP
Cervus_elaphus_hippelaphus   QAIQLLGKMKTLASPFFLAVGYHKPHIPFRYPKEFQKLYPLENVSLAPDP
Desmodus_rotundus            QAIQLLEKMKTSASPFFLAVGYHKPHIPFRYPKEFQKLYPLENITLAPDP
Myotis_lucifugus             KAIQLLEKMKTSSSPFFLAVGYHKPHIPFRYPKEFQKLYPLENITLAPDP
Myotis_brandtii              KAIQLLEKMKTSSSPFFLAVGYHKPHIPFRYPKEFQKLYPLENITLAPDP
Myotis_davidii               KAIQLLEKMKTSSSPFFLAVGYHKPHIPFRYPKEFQKLYPLENITLAPDP
Pteropus_alecto              QAIRLLEKMTSSASPFFLAVGYHKPHIPFRYPKEFQKLYPLENITLAPDP
Felis_catus                  QAIRLLEKMKTSASPFFLAVGYHKPHIPFRYPKEFQKLYPLENITLAPDP
Ailuropoda_melanoleuca       QAIRLLEKTKTSARPFFLAVGYHKPHIPFRYPKEFQKLYPLENITLAPDP
Canis_lupus_familiaris       QAIRLLEKTKTSTRPFFLAVGYHKPHIPFRYPKEFQKLYPLENITLAPDP
Neovison_vison               QAIRLLEKMKTSAHPFFLAVGYHKPHIPFRYPKEFQKLYPLENITLAPDP
                             :** ** * .:   ****.:***********************::****.
```

```
                               301                                              350
Homo_sapiens                   EVPDGLPPVAYNPWMDIRQREDVQALNISVPYGPIPVDF-QRKIRQSYFA
Pan_troglodytes                EVPDGLPPVAYNPWMDIRQREDVQALNISVPYGPIPVDF-QRKIRQSYFA
Gorilla_gorilla_gorilla        EVPDGLPPVAYNPWMDIRQREDVQALNISVPYGPIPVDFQQRKIRQSYFA
Nomascus_leucogenys            EVPDGLPPVAYNPWMDIRQREDVQALNISVPYGPIPVDF-QRKIRQSYFA
Pongo_abelii                   EVPDGLPPVAYNPWMDIRQREDVQALNISVPYGPIPVDF-QQKIRQSYFA
Macaca_mulatta                 EVPDGLPPVAYNPWMDIRQREDVQALNISVPYGPIPVEF-QRKIRQSYFA
Macaca_fascicularis            EVPDGLPPVAYNPWMDIRQREDVQALNISVPYGPIPVEF-QRKIRQSYFA
Papio_anubis                   EVPDGLPPVAYNPWMDIRQREDVQALNISVPYGPIPVEF-QRKIRQSYFA
Chlorocebus_sabaeus            EVPDGLPPVAYNPWMDIRQREDVQALNISVPYGPIPVEF-QRKIRQSYFA
Callithrix_jacchus             EVPDGLPPVAYNPWMDIRQREDVQALNISVPYGPIPVDF-QRKIRQSYFA
Neotoma_lepida                 QVPDGLPPVAYNPWMDIREREDVQALNISVPYGPISVDF-QRKIRQSYFA
Mesocricetus_auratus           QVPDGLPPVAYNPWMDIREREDVQALNISVPYGPIPVDF-QRKIRQSYFA
Rattus_norvegicus              HVPDSLPPVAYNPWMDIREREDVQALNISVPYGPIPVDF-QRKIRQSYFA
Mus_musculus                   HVPDSLPPVAYNPWMDIREREDVQALNISVPYGPIPEDF-QRKIRQSYFA
Dipodomys_ordii                QVPGGLPPVAYNPWMDIRQREDVKALNISVPYGPIPVDF-QQKIRQSYFA
Fukomys_damarensis             EVPDGLPAVAYNPWMDIRQREDVQALNISIPYGPIPVDF-QRKIRQSYFA
Heterocephalus_glaber          EVPDSLPAVAYNPWMDIRQREDVQALNISVPYGPIPVDF-QRKIRQSYFA
Cavia_porcellus                EVPDSLPAVAYNPWMDIRQREDVQALNISVPYGPIPVDF-QRKIRQSYFA
Bos_taurus                     QVPTGLPLVAYNPWMDLRQREDIQALNLSVPYGPIPADF-QRKIRQSYFA
Bos_mutus                      QVPTGLPLVAYNPWMDLRQREDIQALNLSVPYGPIP--F-QRKIRQSYFA
Cervus_elaphus_hippelaphus     QVPAGLPLVAYNPWMDLRQREDVQALNLSVPYGPIPADF-QRKIRQSYFA
Desmodus_rotundus              QVPAGLPPVAYNPWMDIRLREDIQALNLSVPYGPIPVDF-QRKIRQSYFA
Myotis_lucifugus               QVPPGLPPVAYNPWMDIRQREDVEALNLSVPYGSIPVDF-QRKIRQSYFA
Myotis_brandtii                QVPPGLPPVAYNPWMDIRQREDVEALNLSVPYGSIPVDF-QRKIRQSYFA
Myotis_davidii                 QVPPGLPPVAYNPWMDIRQREDVEALNLSVPYGSIPVDF-QRKIRQSYFA
Pteropus_alecto                QVPAGLPPVAYNPWMDIRQREDVKALNLSVPYGPIPVDF-QRKIRQSYFA
Felis_catus                    QVPAGLPPVAYNPWMDIRQREDVQALNLSVPYGPIPVDF-QRKIRQSYFA
Ailuropoda_melanoleuca         QVPAGLPPVAYNPWMDIRQREDVQALNLSVPYGPIPVDF-QRKIRQSYFA
Canis_lupus_familiaris         EVPAGLPPVAYNPWMDIRQREDVQALNLSVPYGPIPVDF-QRKIRQSYFA
Neovison_vison                 QVPAGLPPVAYNPWMDIRQREDVQALNLSVPYGPIPVDF-QRKIRQSYFA
                               .** .** ********:* ***::***:*:***.*.  * *:********

                               351                                              400
Homo_sapiens                   SVSYLDTQVGRLLSALDDLQLANSTIIAFTSDH--GWALGEHGEWAKYSN
Pan_troglodytes                SVSYLDTQVGRLLSALDDLQLANSTIIAFTSDH--GWALGEHGEWAKYSN
Gorilla_gorilla_gorilla        SVSYLDTQVGRLLSALDDLQLANSTIIAFTSDH--GWALGEHGEWAKYSN
Nomascus_leucogenys            SVSYLDTQVGRLLSALDDLQLANSTIIAFTSDH--GWALGEHGEWAKYSN
Pongo_abelii                   SVSYLDTQVGRLLSTLDDLQLANSTIIAFTSDH--GWALGEHGEWAKYSN
Macaca_mulatta                 SVSYLDTQVGRLLSALDDLQLANSTIVAFTSDH--GWALGEHGEWAKYSN
Macaca_fascicularis            SVSYLDTQVGHLLSALDDLQLANSTIVAFTSDH--GWALGEHGEWAKYSN
Papio_anubis                   SVSYLDTQVGRLLSALDDLQLANSTIVAFTSDH--GWALGEHGEWAKYSN
Chlorocebus_sabaeus            SVSYLDTQVGRLLSALDDLQLANSTIVAFTSDH--GWALGEHGEWAKYSN
Callithrix_jacchus             SVSYLDTQVGHLLSALDDLQLANSTIVAFTSDH--GWALGEHGEWAKYSN
Neotoma_lepida                 SVSYLDTQVGHLLSALDDLHLANNTIIAFTSDH--GWALGEHGEWAKYSN
Mesocricetus_auratus           SVSYLDTEVGHLLSALDDLHLANNTIIAFTSDH--GWALGEHGEWAKYSN
Rattus_norvegicus              SVSYLDTQVGHLLSALDDLRLAHNTIIAFMSDH--GWALGEHGEWAKYSN
Mus_musculus                   SVSYLDTQVGHVLSALDDLRLAHNTIIAFTSDH--GWALGEHGEWAKYSN
Dipodomys_ordii                SVSYLDTQVGHLLGALDDLELANRTIIAFMSDH--GWSLGEHGEWAKYSN
Fukomys_damarensis             SVSYLDTQVGRLLSALDDLQLANSTIIAFTSDH--GWALGEHGEWAKYSN
Heterocephalus_glaber          SVSYLDTQVGRLLSALDDLQLANSTIIALTSDH--GWALGEHGEWAKYSN
Cavia_porcellus                SVSYLDTQVGHLLSALDDLQLANNTIIAFTSDH--GWALGEHGEWAKYSN
Bos_taurus                     CVSYLDTQVGRLLSALDDLQLASSTIVAFTSDH--GWALGEHGEWAKYSN
Bos_mutus                      CVSYLDTQVGRLLSALDDLQLASSTIVAFTSDH--GWALGEHGEWAKYSN
Cervus_elaphus_hippelaphus     CVSYLDTQVGHLLSALDDLQLASSTIVALTSDH--GWALGEHGEWAKYSN
Desmodus_rotundus              SVSYLDTQVGHLLSALDDFHLANSTIVAFTSDH--GWSLGEHGEWAKYSN
Myotis_lucifugus               SVSYMDTQVGHLLSALDDLQLANSTIVAFTSDHEPGWALGEHGEWAKYSN
Myotis_brandtii                SVSYMDTQVGHLLSALDDLQLANSTIVAFTSDH--GWALGEHGEWAKYSN
Myotis_davidii                 SVSYMDSQVGHLLSALDDLQLANSTIVAFTSDH--GWALGEHGEWAKYSN
Pteropus_alecto                SVSYLDTQVGHLLSSLDDLHLANNTIVAFTSDH--GWALGEHGEWAKYSN
Felis_catus                    SVSYLDTQVGHLLSALDDLQLTNSTIIAFASDH--GWALGEHGEWAKYSN
Ailuropoda_melanoleuca         SISYLDTQVGHLLSALDDLELANSTIIVFVSDH--GWALGEHGEWAKYSN
Canis_lupus_familiaris         SISYLDTQVGHLLSALDDLQLANSTIIVFASDH--GWALGEHGEWAKYSN
Neovison_vison                 SVSYLDTQVGHLLSALDDLQLANSTIIAFVSDH--GWALGEHGEWAKYSN
                               .:**:*::**::*.:***:.*:  **:.: ***  **:************
```

```
                            401                                            450
Homo_sapiens                FDVATHVPLIFYVPGRTASLPEAGEKLFPYLDPFDSASQLMEPGRQSMDL
Pan_troglodytes             FDVATHVPLMFYVPGRTASLPEAGEKLFPYLDPFDSASQLMEPGRQSMDL
Gorilla_gorilla_gorilla     FDVATHVPLMFYVPGRTASLPEAGEKLFPYLDPFDSASQLMEPGRQSMDL
Nomascus_leucogenys         FDVATHVPLMFYVPGRTASLPEAGEKLFPYLDPFDSASELMEPGRQSMDL
Pongo_abelii                FDVATHVPLMFYVPGRTASLPEAGEKLFPYLDPFDSASELMEPGRQSMDL
Macaca_mulatta              FDVATHVPLMFYVPGRTASLPEAGEKLFPYLDPFDSASELMEPGRQSMDL
Macaca_fascicularis         FDVATHVPLMFYVPGRTASLPEAGEKLFPYLDPFDSASELMEPGRQSMDL
Papio_anubis                FDVATHVPLMFYVPGRTASLPEAGEKLFPYLDPFDSASELMEPGRQSMDL
Chlorocebus_sabaeus         FDVATHVPLMFYVPGRTASLPEAGEKLFPYLDPFDSASELMEPGRQSMDL
Callithrix_jacchus          FDVATRVPLMFYVPGRTASLPEADEKLFPYVDPFHSASELMEPGRQSMDL
Neotoma_lepida              FDVTTRVPLMFYVPGKTAPLPGVDEKLFPYQDPFNLTSKLMDPGRHIVDL
Mesocricetus_auratus        FDVTTRVPLMFYVPGKTAPLPGPGEKLFPYQDPFDPTSKSMDSGRHVVDL
Rattus_norvegicus           FDVTTRVPLMFYVPGKTALLPAAGEKLFPYQDPFDPASELMGAGRRTEDL
Mus_musculus                FDVATRVPLMLYVPGRTAPLPAAGQKLFPYRDPFDPASDWMDAGRHTEDL
Dipodomys_ordii             FDVTTRVPLMFYVPGKTAPLPEAGQKLFPYRDPFDLASQWMAPGRQSWDL
Fukomys_damarensis          FDVTTRVPLMFYVPGRTTSFPEAGEMLFPYLDPFDSASQLMEPGRQATDL
Heterocephalus_glaber       FDVTTRVPLMFYVPGRTTSFLEAGEMLFPYLDPFDSASELMEPGRQTTDL
Cavia_porcellus             FDVTTRVPLMFYVPGRTTSFPEAGKMLFPYLDPFDSASQSMEPGMQTTDL
Bos_taurus                  FDVTTRVPLMFYVPGRTAPLPVEGEKFFPYLDPFDSVSEVPEPGMEMQNL
Bos_mutus                   FDVTTRVPLMFYVPGRTAPLPVEGEKFFPYLDPFDSVSEVPEPGRQTGDL
Cervus_elaphus_hippelaphus  FDVTTRVPLMFYVPGRTAPLPVEGEKFFPYLDPFDSISEAPEPGRQTEDL
Desmodus_rotundus           FDVATHVPLMFYVPGRTAVLPEAGEKLFPYINPFDPASELMEPGQQAVDL
Myotis_lucifugus            FDVTTHVPLMFYVPGRTAPLPEAGEKLFPYIDPFDSISELMEPGRQVQDL
Myotis_brandtii             FDVTTHVPLMFYVPGRTAPLPEAGEKLFPYIDPFDSISELMEPGRQVQDL
Myotis_davidii              FDVTTHVPLMFYVPGRTALLPEAGEKLFPYIDPFDSISELMEPGRQVEDL
Pteropus_alecto             FDITTRVPLMFYVPGRTAPLPEAGKKLFPYIDPFDS-SELMEPGRRAEDL
Felis_catus                 FDVATRVPLMFYVPGRTAPLPEAGEELFPYIDPFDSVLESMEPGRRVMDL
Ailuropoda_melanoleuca      FDVTTRVPLMFYVPGRTAPLPEAGEKLFPFVDPFDSVLERMEPGRQVMDL
Canis_lupus_familiaris      FDITTRVPLMFYVPGRTAPLPEAGEKLFPYIDPFSSVQELMEPGRQVTDL
Neovison_vison              FDVATRVPLMFYVPGRTAPLLEAGEKLFPYIDPFDSIVELMEPGRQVVDL
                            **::*:***::****:*: :   .: :**: :**    .   .* .  :*

                            451                                            500
Homo_sapiens                VELVSLFPTLAGLAGLQVPPRCPVPSFHVELCREGKNLLKHFRFRDLEED
Pan_troglodytes             VELVSLFPTLAGLAGLQAPPRCPVPSFHVELCREGKNLLKHFRFRDLEED
Gorilla_gorilla_gorilla     VELVSLFPTLAGLAGLQVPPRCPVPSFHVELCREGKNLLKHFRFRDLEED
Nomascus_leucogenys         VELVSLFPTLAGLAGLQVPPRCPVPSFHVELCREGKNLLKHFRFRDLEED
Pongo_abelii                VELVSLFPTLAGLAGLQVPPRCPVPSFHVELCREGKNLLKHFRFRDLEED
Macaca_mulatta              VELVSLFPTLAGLAGLQVPPRCPVPSFHVELCREGKNLLKHFRFRDLEED
Macaca_fascicularis         VELVSLFPTLAGLAGLQVPPRCPVPSFHVELCREGKNLLKHFRFRDLEED
Papio_anubis                VELVSLFPTLAGLAGLQVPPRCPVPSFHVELCREGKNLLKHFRFRDLEED
Chlorocebus_sabaeus         VELVSLFPTLAGLAGLQVPPRCPVPSFHVELCREGKNLLKHFRFRDLEED
Callithrix_jacchus          VELVSLFPTLAGLAGLQVPPRCPVPSFHVELCREGKSLLKHFRFHGLEED
Neotoma_lepida              VELVSLFPTLAGLAGLQVPPRCPIPSFHVELCREGQNLQKHLQLRDLEED
Mesocricetus_auratus        VELVSLFPTLAELSGLQVPPRCPIPSFHVELCREGQSLQKHLQLHDFEEE
Rattus_norvegicus           VELVSLFPTLAGLAGMPVPPWCPIPSFHVELCREGHNLQRYLQLHDLEED
Mus_musculus                VELVSLFPTLAGLAGLPVPPRCPIPSFHVELCREGQNLQKHLQLHDLEEE
Dipodomys_ordii             VELMSLFPTLAGLAGLQVPPRCPIPSFHIELCREGQSLLNHFRFHDWEED
Fukomys_damarensis          VELVSLFPTLTGLAGLHVPPQCPVPSFDVELCREGEDLLKHFRSRDLEED
Heterocephalus_glaber       VELVSLFPTLTGLAGLHVPPQCPVPSFDVEVCREGDNLLKHFRSRDLEED
Cavia_porcellus             VELVSLFPTLASLAGLPILPPCPVPSFHVELCREGENLLKHFGSHDLEED
Bos_taurus                  LHALSSFMSLIGLSFLLRPRHCKDPK-RLELWPRGRNLLKLFEDHGEEEE
Bos_mutus                   VELLSLFPTLAGLAGLRVPPRCPIPSFHMELCREGRNLLKLFEDHGEEEE
Cervus_elaphus_hippelaphus  VELLSLFPTLTGLAGLRVPPRCPIPSFHMELCREGRNLLKLFEGRGEEEE
Desmodus_rotundus           VELVSLFPTLTGLAGLHVPPRCPIPSFHVELCREGQSLLKHFRFHDLEED
Myotis_lucifugus            VELVSLFPTLAGLAGLHVPPRCPSPSFHVELCREGHSLLNHFQFHDLEED
Myotis_brandtii             VELVSLFPTLAGLAGLHVPPRCPSPSFHVELCREGHSLLNHFQFHDLEED
Myotis_davidii              VELVSLFPTLAGLAGLHVPPRCPSPSFHVELCREGQSLLNHFQFHDLEED
Pteropus_alecto             VELVSLFPTLAGLAGLRVPPRCPVPSFHVELCREGQSLGKHFRPHDLEED
Felis_catus                 VELLSLSPTLAGLAGLHVPPRCPVPSFHVQLCREGQNLGKHFQLQDLGED
Ailuropoda_melanoleuca      VELLSLSPTLAGLAGLHVPPRCPVPSFHVKLCREGQSLVKHFRFQDLEDD
Canis_lupus_familiaris      VELLSLSPTLAGLAGLHVPPRCPVPSFHVELCREGQNLMKHFQVEDVEGD
Neovison_vison              VELLSLFPTLAGLAGLRVPPRCPVPSFRVAQCREGQSLVKYFRFQDLDED
                            :. :*   :*  *: :     *  *.  :    .* .* . :  ..   :
```

```
                            501                                            550
Homo_sapiens                PYLPGNPRELIAYSQYPRPSDIPQWNSDKPSLKDIKIMGYSIRTIDYRYT
Pan_troglodytes             PYLPGNPRELIAYSQYPRPSDIPQWNSDKPSLKDIKIMGYSIRTIDYRYT
Gorilla_gorilla_gorilla     PYLPGNPRELIAYSQYPRPADIPQWNSDKPSLKDIKIMGYSIRTIDYRYT
Nomascus_leucogenys         PYLPGNPRELIAYSQYPRPADIPQWNSDKPSLKDIKIMGYSIRTIDYRYT
Pongo_abelii                PYLPGNPRELIAYSQYPRPADIPQWNSDKPSLKDIKIMGYSIRTIDYRYT
Macaca_mulatta              PYLPGNPRELIAYSQYPRPADFPQWNSDKPSLKDIKIMGYSIRTIDYRYT
Macaca_fascicularis         PYLPGNPRELIAYSQYPRPADFPQWNSDKPSLKDIKIMGYSIRTIDYRYT
Papio_anubis                PYLPGNPRELIAYSQYPRPADFPQWNSDKPSLKDIKIMGYSIRTIDYRYT
Chlorocebus_sabaeus         PYLPGNPRELIAYSQYPRPADFPQWNSDKPNLKDIKIMGYSIRTIDYRYT
Callithrix_jacchus          PYLPGNPRELIAYSQYPRPADFPQWNSDKPSLKYIKIMGYSIRTVDYRYT
Neotoma_lepida              PHFLGNPRELIAYSQYPRPADSPQWNSDKPSLKDIKIMGYSIRTVDYRYT
Mesocricetus_auratus        LHFLGNPRESIAYSQYPRPADSPQWNSDKPSLKDIKIMGYSIRTIDYRYT
Rattus_norvegicus           PYLFGNPRELIAYSQYPRPADFPQWNSDKPSLKDIKVMGYSIRTIDYRYT
Mus_musculus                PDLFGNPRELIAYSQYPRPADFPQWNSDKPSLNDIKVMGYSIRTVDYRYT
Dipodomys_ordii             PHLHGNPHESVAYSQYPRPADSPQWNSDKPSLKDIKIMGYSIRTIDYRYT
Fukomys_damarensis          PVLHDNPRELVAYSQYPRPADSPQWNSDKPSLKDIKIMGYSIRTIDYRYT
Heterocephalus_glaber       PDLHDNPRESVAYSQYPRPADSPQWNSDKPSLKDIKIMGYSIRTVDYRYT
Cavia_porcellus             PELRGNPRELVAYSQYPRPADTPQWNSDNPSLKDIKVMGYSIRTIDYRYT
Bos_taurus                  PHVRSNPRELVAYSQYPRPADSPQWNSDKPSLEDIKVMGYSIRTVDYRYT
Bos_mutus                   PHVRSNPRELVAYSQYPRPADSPQWNSDKPSLEDIKVMGYSIRTVDYRYT
Cervus_elaphus_hippelaphus  PHVRGNPRELVAYSQYPRPADSPQWNSDKPSLEDIKVMGYSIRTVDYRYT
Desmodus_rotundus           LTLHDNPHESIAYSQYPRPANYPQWNSDKPSLKDIKVMGYSIRTTDYRYT
Myotis_lucifugus            LTLRGNPRESIAYSQYPRPADYPQWNSDKPSLKDIKFMGYSIRTIDYRYT
Myotis_brandtii             LTLRGNPRESIAYSQYPRPADYPQWNSDKPSLKDIKFMGYSIRTIDYRYT
Myotis_davidii              LTHRGNPRESIAYSQYPRPADYPQWNSDKPSLKDIKFMGYSIRTIDYRYT
Pteropus_alecto             PALHGDPRESIAYSQYPRPADSPQWNSDKPSLKDIKVMGYSIRTIDYRFT
Felis_catus                 PHLRGNPRESIAYSQYPRPADYPQWNSDKPSLKDIKVMGYSIRTIDYRYT
Ailuropoda_melanoleuca      PSLRGNPRESIAYSQYPRPADSPQWNSDKPSLKDIKIMGYSIRTIDYRYT
Canis_lupus_familiaris      PHLRGNPRESIAYSQYPRPADSPQWNSDKPSLKDIKVMGYSIRTIDYRYT
Neovison_vison              QYLRGNPRESVAYSQYPRPADSPQWNSDKPSLKDIKVMGYSIRTVDYRYT
                                .:*:* :********:: ******:*.*: **.******* ***:*
```

```
                           551                                                   606
Homo_sapiens               VWVGFNPDEFLANFSDIHAGELYFVDSDPLQDHNMYNDSQGGDLFQLLMP------
Pan_troglodytes            VWIGFNPDEFLANFSDIHAGELYFVDSDPLQDHNMYNDSQGGDLFQLLMP------
Gorilla_gorilla_gorilla    VWVGFNPDEFLANFSDIHAGELYFVDSDPLQDHNMYNDSQGGDLFQLLMP------
Nomascus_leucogenys        VWVGFSPDEFLANFSDIHAGELYFVDSDPLQDHNMYNDSQGGDLFQLLMP------
Pongo_abelii               VWVGFNPDEFLANFSDIHAGELYFVDSDPLQDHNMYNDSQGGDLFQLLMP------
Macaca_mulatta             VWVGFNPDEFLANFSDIHAGELYFVDSDPLQDHNMYNDSQGGDLFQLLMP------
Macaca_fascicularis        VWVGFNPDEFLANFSDIHAGELYFVDSDPLQDHNMYNDSQGGDLFQLLMP------
Papio_anubis               VWVGFNPDEFLANFSDIHAGELYFVDSDPLQDHNMYNDSQGGDLFQLLMP------
Chlorocebus_sabaeus        VWVGFNPDEFLANFSDIHAGELYFVDSDPLQDHNMYNDSQGGDLFQLLMP------
Callithrix_jacchus         VWVGFNPDEFLANFSDIHAGELYFVDSDPLQDHNMYNDSQGGELFQSLMP------
Neotoma_lepida             VWVGFNPAEFLANFSDIHAGELYFVDSDPLQDHNVYNDSQHGGLPYSLKP------
Mesocricetus_auratu        VWVGFNPAEFLANFSDIHAGELYFVDSDPLQDHNVYNDSQHGGLPHSLNP------
Rattus_norvegicus          VWVGFSPTEFLANFSDIHAGELYFVDSDPLQDHNVYNDSQHGGLLLSLRP------
Mus_musculus               VWVGFDPSEFLANFSDIHAGELYFVDSDPLQDHNVYNDSQHGGLLHSLRP------
Dipodomys_ordii            VWVGFNPDEFLANFTDIHAGELYFVDSDPLQDHNVYNDSQHREFLQSLIP------
Fukomys_damarensis         VWVGFDPDEFLANFSDIHAGELYFVDSDPLQDHNIYNYSQHGEIPPSLMP------
Heterocephalus_glaber      VWVGFDPDEFLANFSDIHAGELYFVDSDPLQDHNIYNYSQHGEIPPSLMP------
Cavia_porcellus            VWVGFDPHEFLANFSDIHAGELYFVDSDPLQDHNIYNYSQHGEIPPSLMP------
Bos_taurus                 VWVGFNPQEFLANFSDIHAGELYFVASDPLQDHNVYNDSQGGTLPQSFMP------
Bos_mutus                  VWVGFNPQEFLANFSDIHAGELYFVASDPLQDHNVYNDSQGGTLPQSFMP------
Cervus_elaphus_hippelaphus VWVGFNPQEFLANFSDIHAGELYFVASDPLQDHNVYNDSLGETLPQSFIP------
Desmodus_rotundus          VWVGFNPHEFLANFSDVHAGELYFVDTDPLQDHNVYNDFQGGDLLQSLIP------
Myotis_lucifugus           VWVSFNPHEFLANFSDVHAGELYFVDSDPLQDYNMYNETQGGDLPQLLTP------
Myotis_brandtii            VWVSFNPHEFLANFSDVHAGELYFVDSDPLQDHNMYNETQGGDLPQLLTP------
Myotis_davidii             VWVSFNPHEFLANFSDVHAGELYFVDSDPLQDYNMYNETQGGDLPQLLTP------
Pteropus_alecto            VWVGFSPHEFLANFSDVHAGELYFVDSDPLQDHNMYNDTQGEDLPRLLAP------
Felis_catus                VWVGFDPREFLANFSDVHAGELYFVDSDPLQDHNMYNDSQGRDLLQSLMP------
Ailuropoda_melanoleuca     VWVGFSPHEFLANFSDVHAGELYFVDSDPLQDHNMYNDSQGRDLLRSLMP------
Canis_lupus_familiaris     VWVGFSPHEFLANFSDVHAGELYFVDSDPLQDHNMYNDSQGKDLLRAFDAIFLANH
Neovison_vison             VWVGFNPREFLANFSDVHAGELYFIDSDPLQDHNMYNGSWGRDLLQSLLP------
                           **:.*.* ******:*:*******: :*****:*:**      :   : .
```

B

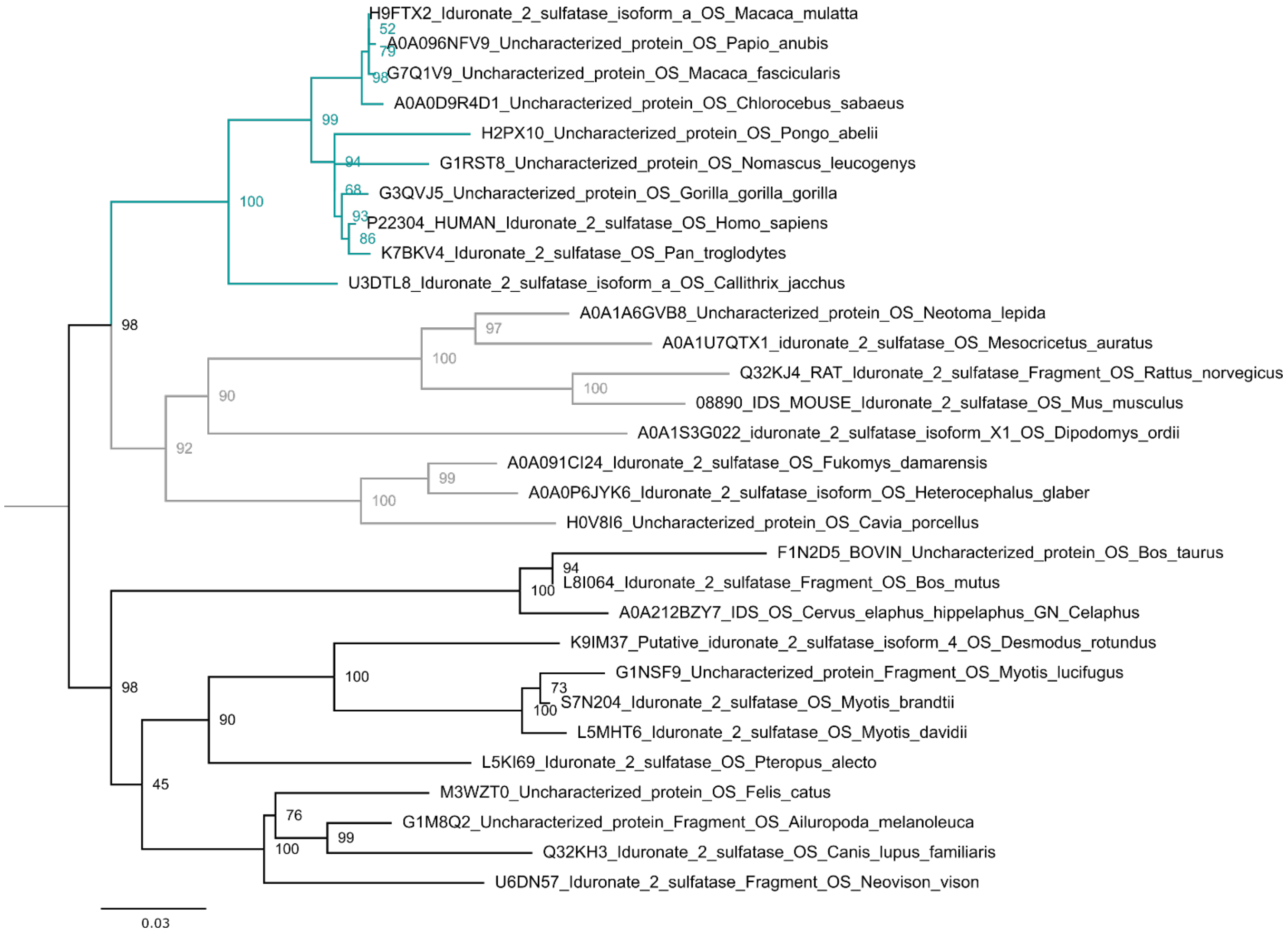

Figure S2: Multiple sequence alignment and phylogenetic tree of mammalian IDS homologues, related to Figure 1. A) Full-length multiple sequence alignment of 30 mammalian IDS homologues that was used for ancestral sequence reconstruction. The positions that were excluded by trimming (as described in the Transparent Methods section) are shaded in grey. **B)** Maximum Likelihood tree of the same set of sequences created with IQTREE (Nguyen et al., 2015). Bootstrap values are shown for 1000 replicates and the colored clades are primates (green) and rodents (grey).

```
hIDS            MPPPRTGRGLLWLGLVLSSVCVALGSETQANSTTDALNVLLIIVDDLRPSLGCYGDKLVR
IDS_A1          MPPPRTGRGLLWLGLVLSSVCVALGSETQANSTTDALNVLLIIVDDLRPSLGCYGDKLVR
IDS_A2          MPPPRTGRGLLWLGLVLSSVCVALGSETQANSTTDALNVLLIIVDDLRPSLGCYGDKLVR
IDS_A3          MPPPRTGRGLLWLGLVLSSVCVALGSETQANSTTDALNVLLIIVDDLRPSLGCYGDKLVR
                ************************************************************

hIDS            SPNIDQLASHSLLFQNAFAQQAVCAPSRVSFLTGRRPDTTRLYDFNSYWRVHAGNFSTIP
IDS_A1          SPNIDQLASHSLLFQNAFAQQAVCAPSRVSFLTGRRPDTTRLYDFNSYWRVHAGNFSTIP
IDS_A2          SPNIDQLASHSLLFQNAFAQQAVCAPSRVSFLTGRRPDTTRLYDFNSYWRVHAGNFSTIP
IDS_A3          SPNIDQLASHSLLFQNAFAQQAVCAPSRVSFLTGRRPDTTRLYDFNSYWRVHAGNFSTIP
                ************************************************************

hIDS            QYFKENGYVTMSVGKVFHPGISSNHTDDSPYSWSFPPYHPSSEKYENTKTCRGPDGELHA
IDS_A1          QYFKENGYVTMSVGKVFHPGISSNHTDDSPYSWSFPPYHPSSEKYENTKTCRGPDGELHA
IDS_A2          QYFKENGYVTMSVGKVFHPGISSNHSDDSPYSWSFPPYHPSSEKYENTKTCRGPDGELHA
IDS_A3          QYFKENGYVTMSVGKVFHPGISSNHSDDSPYSWSFPPYHPSSEKYENTKTCRGPDGELHA
                *************************:**********************************

hIDS            NLLCPVDVLDVPEGTLPDKQSTEQAIQLLEKMKTSASPFFLAVGYHKPHIPFRYPKEFQK
IDS_A1          NLLCPVDVVDVPEGTLPDKQSTEQAIQLLEKMKTSASPFFLAVGYHKPHIPFRYPKEFQK
IDS_A2          NLLCPVDVVDVPEGTLPDKQSTEQAIRLLEKMKTSASPFFLAVGYHKPHIPFRYPKEFQK
IDS_A3          NLLCPVDVADVPEGTLPDKQSTEQAIRLLEKMKTSASPFFLAVGYHKPHIPFRYPKEFQK
                ******** *****************:*********************************

hIDS            LYPLENITLAPDPEVPDGLPPVAYNPWMDIRQREDVQALNISVPYGPIPVDFQRKIRQSY
IDS_A1          LYPLENITLAPDPEVPDGLPPVAYNPWMDIRQREDVQALNISVPYGPIPVDFQRKIRQSY
IDS_A2          LYPLENITLAPDPEVPDGLPPVAYNPWMDIRQREDVQALNISVPYGPIPVDFQRKIRQSY
IDS_A3          LYPLENITLAPDPQVPDGLPPVAYNPWMDIRQREDVQALNISVPYGPIPVDFQRKIRQSY
                *************:**********************************************

hIDS            FASVSYLDTQVGRLLSALDDLQLANSTIIAFTSDHGWALGEHGEWAKYSNFDVATHVPLI
IDS_A1          FASVSYLDTQVGRLLSALDDLQLANSTIVAFTSDHGWALGEHGEWAKYSNFDVATHVPLM
IDS_A2          FASVSYLDTQVGHLLSALDDLQLANSTIVAFTSDHGWALGEHGEWAKYSNFDVATRVPLM
IDS_A3          FASVSYLDTQVGHLLSALDDLQLANSTIVAFTSDHGWALGEHGEWAKYSNFDVTTRVPLM
                ************:***************:************************:*:***:

hIDS            FYVPGRTASLPEAGEKLFPYLDPFDSASQLMEPGRQSMDLVELVSLFPTLAGLAGLQVPP
IDS_A1          FYVPGRTASLPEAGEKLFPYLDPFDSASELMEPGRQSMDLVELVSLFPTLAGLAGLQVPP
IDS_A2          FYVPGRTASLPEAGEKLFPYLDPFDSASELMEPGRQSMDLVELVSLFPTLAGLAGLQVPP
IDS_A3          FYVPGRTAPLPEAGEKLFPYLDPFDSASELMEPGRQTMDLVELVSLFPTLAGLAGLQVPP
                ********.*******************:*******:***********************

hIDS            RCPVPSFHVELCREGKNLLKHFRFRDLEEDPYLPGNPRELIAYSQYPRPSDIPQWNSDKP
IDS_A1          RCPVPSFHVELCREGKNLLKHFRFRDLEEDPYLPGNPRELIAYSQYPRPADFPQWNSDKP
IDS_A2          RCPVPSFHVELCREGKNLLKHFRFHDLEEDPYLPGNPRELIAYSQYPRPADFPQWNSDKP
IDS_A3          RCPVPSFHVELCREGQNLLKHFRFHDLEEDPHLHGNPRELIAYSQYPRPADSPQWNSDKP
                ***************:********:******:* ***************:* ********

hIDS            SLKDIKIMGYSIRTIDYRYTVWVGFNPDEFLANFSDIHAGELYFVDSDPLQDHNMYNDSQ
IDS_A1          SLKDIKIMGYSIRTIDYRYTVWVGFNPDEFLANFSDIHAGELYFVDSDPLQDHNMYNDSQ
IDS_A2          SLKDIKIMGYSIRTIDYRYTVWVGFNPDEFLANFSDIHAGELYFVDSDPLQDHNMYNDSQ
IDS_A3          SLKDIKIMGYSIRTIDYRYTVWVGFNPDEFLANFSDIHAGELYFVDSDPLQDHNMYNDSQ
                ************************************************************

hIDS            GGDLFQLLMP
IDS_A1          GGDLFQLLMP
IDS_A2          GGDLFQSLMP
IDS_A3          GGDLPQSLMP
                **** * ***
```

Figure S3: Alignment of hIDS and ancestors IDS-A1, IDS-A2 and IDS-A3, related to Figure 2. All ancestral mutations are marked in yellow, except for mutation A354T that is marked in red. Cysteine 84 that is post-translationally modified to formylglycine is marked in cyan and all known glycosylation sites are market in grey. The signal sequence is shown in grey and the sequence that is susceptible to proteolytic cleavage in the lysosome is underlined. The alignment was created using MAFFT.

Table S1: IDS variants that were evaluated in this study, related to Figure 2 and 3.

	# Mutations (compared to hIDS)[a]	% Identity (compared to hIDS)[a]	% Identity (compared to mIDS)[a]
hIDS_A354T	1	99.8	88.2
hIDS_H456R	1	99.8	88.2
hIDS_A354T_H354R	2	99.6	88.4
IDS-A1	6	98.8	88.8
IDS-A1_V329I	5	99.0	89.0
IDS-A2	12	97.7	89.9
IDS-A3	20	96.1	90.3
IDS-A3_V329I	19	96.3	90.5
IDS-A3_T354A	19	96.3	90.5
mIDS	60	88.4	100

[a] Signal sequences are excluded

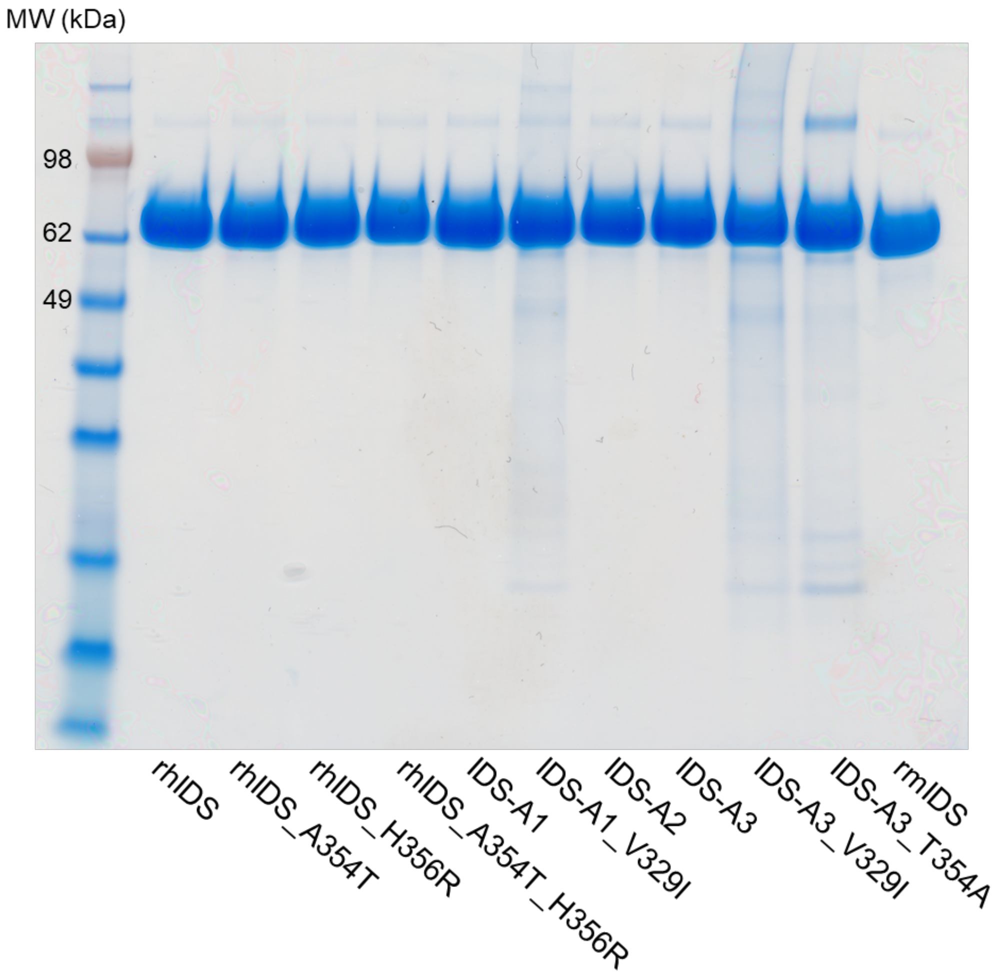

Figure S4: IDS enzymes can be functionally expressed in ExpiCHO, related to Figure 3. SDS-PAGE showing batches of purified IDS enzymes; the molecular weight of glycosylated IDS enzymes is around 76 kDa. The SeeBlue™ Plus2 pre-stained protein standard is shown for reference and 10 µg of protein was loaded for each enzyme.

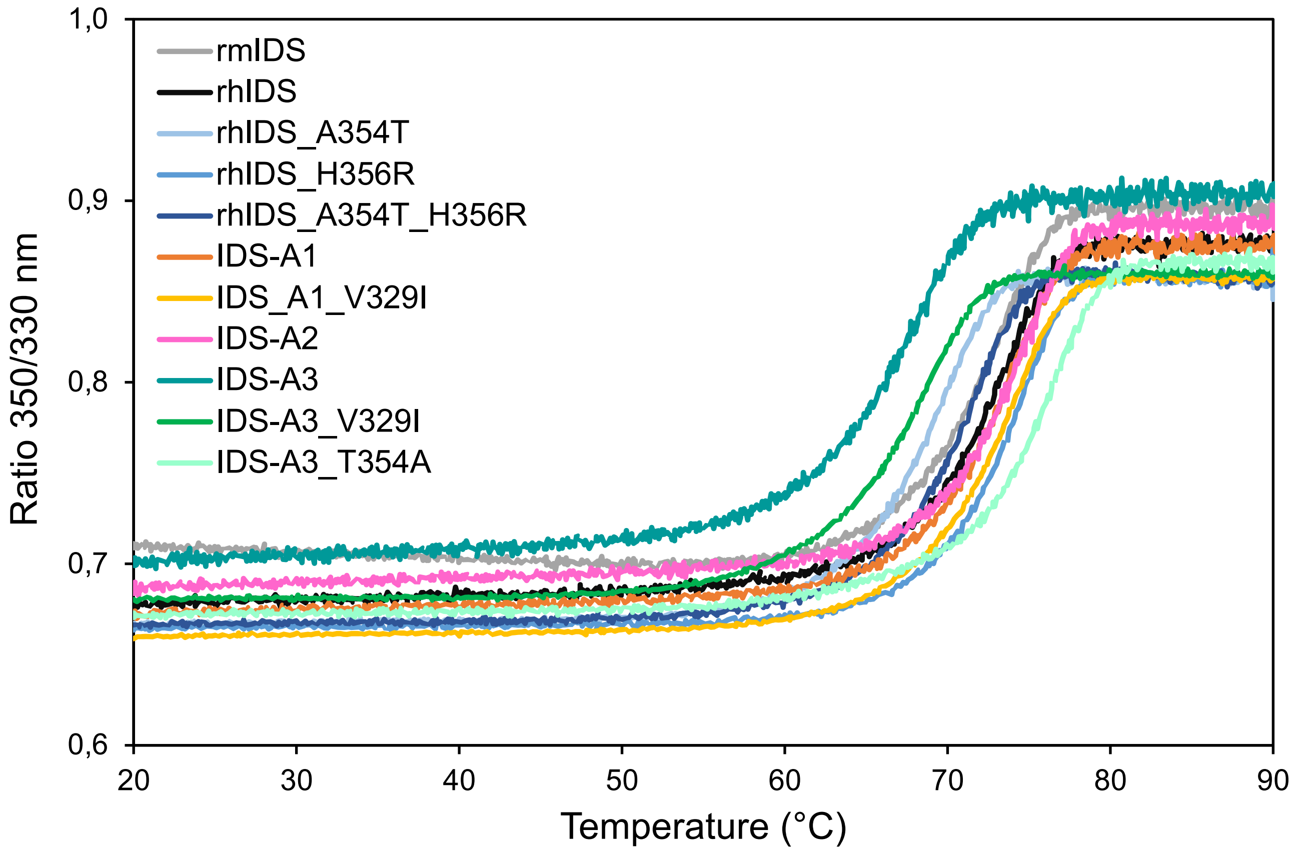

Figure S5: Melting curves of IDS enzymes established by nanoDSF, related to Table 1. The ratio of intrinsic tryptophan fluorescence at 350 over 330 nm was monitored while heating the samples with 1 degree per minute from 20 °C to 90 °C. The melting temperature is determined as the maximum of the first derivative of the ratio.

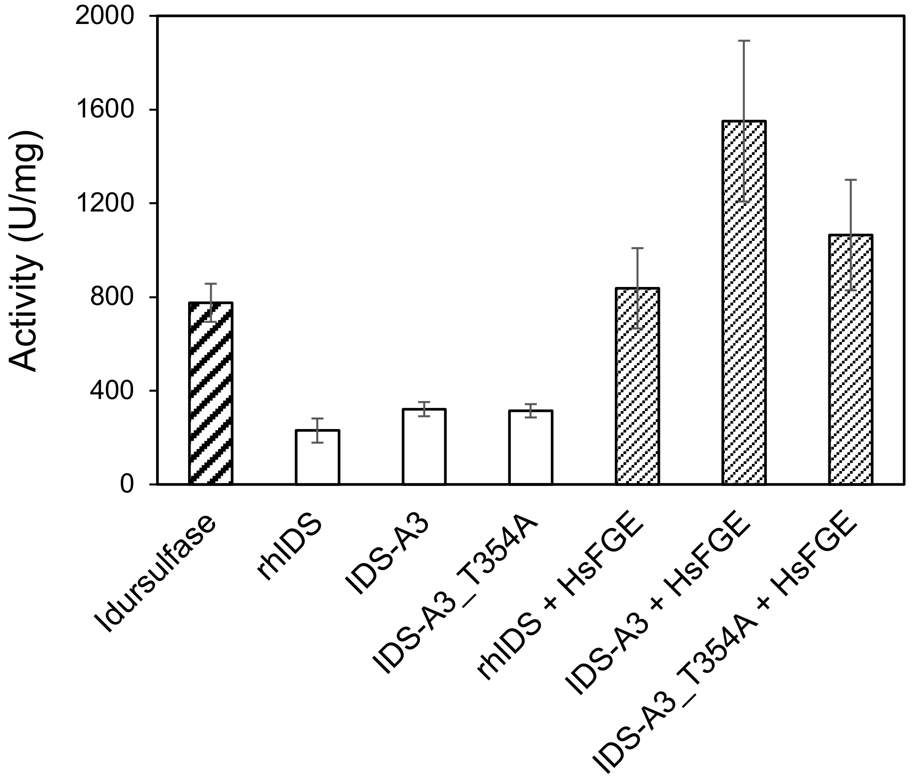

Figure S6: Activity of hIDS, IDS-A3 and variants, related to Figure 3. Comparison of activities of hIDS, IDS-A3 and IDS-A3_T354A with and without co-expression of *Hs*FGE, Idursulfase is included for reference. Average activities are shown for two independent transfection experiments for each variant and error bars show the standard deviations from 6-12 replicates.

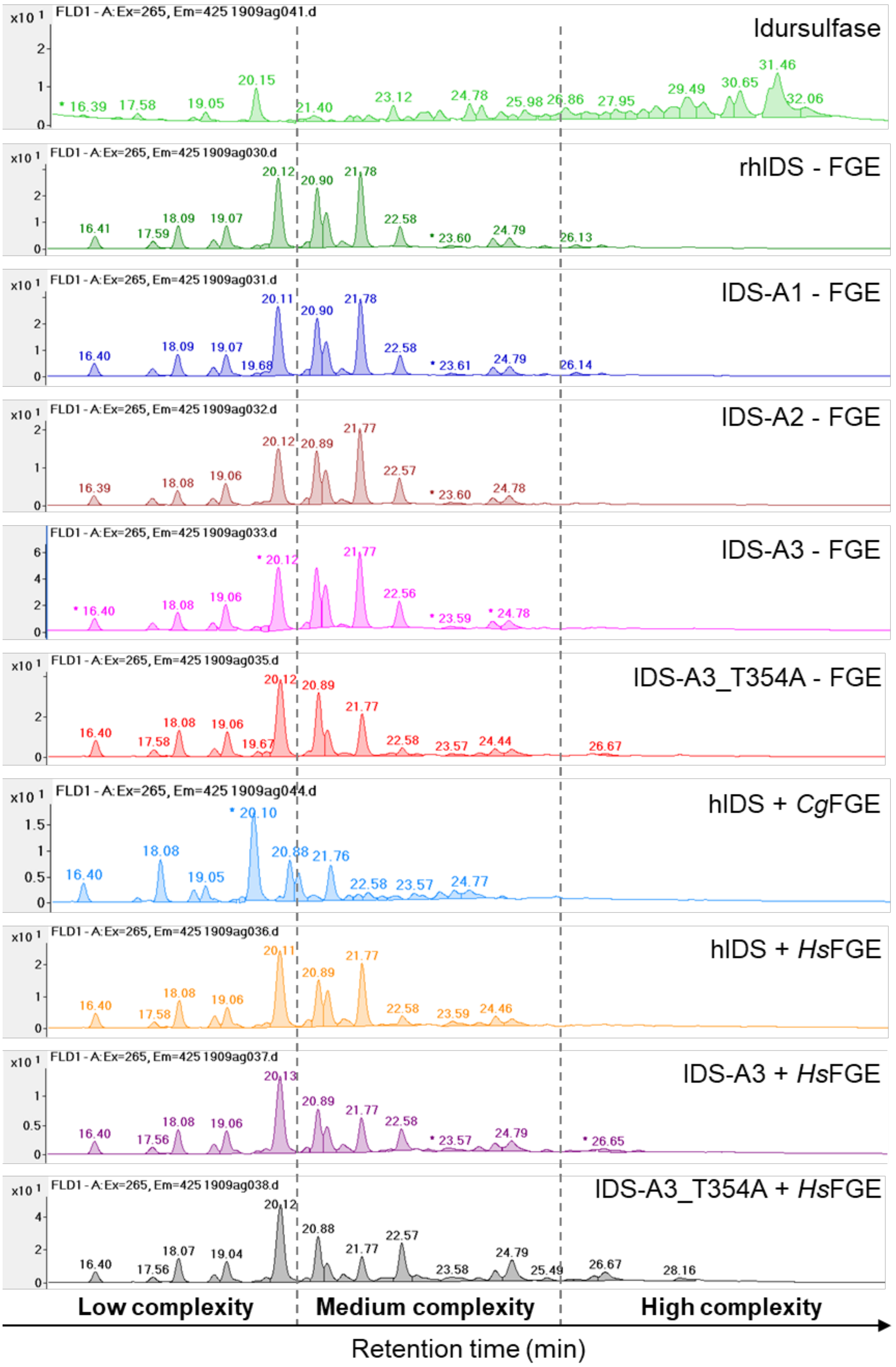

Figure S7: Glycan profiles of IDS enzymes, related to Figure 5. Profiles of released N-glycans as determined by the GlycoWorks *Rapi*Fluor-MS N-Glycan Kit. Glycans were divided into three categories for comparison, based on retention time in LC-FLR-MS: simple, semi-mature and mature glycans (shown on bottom).

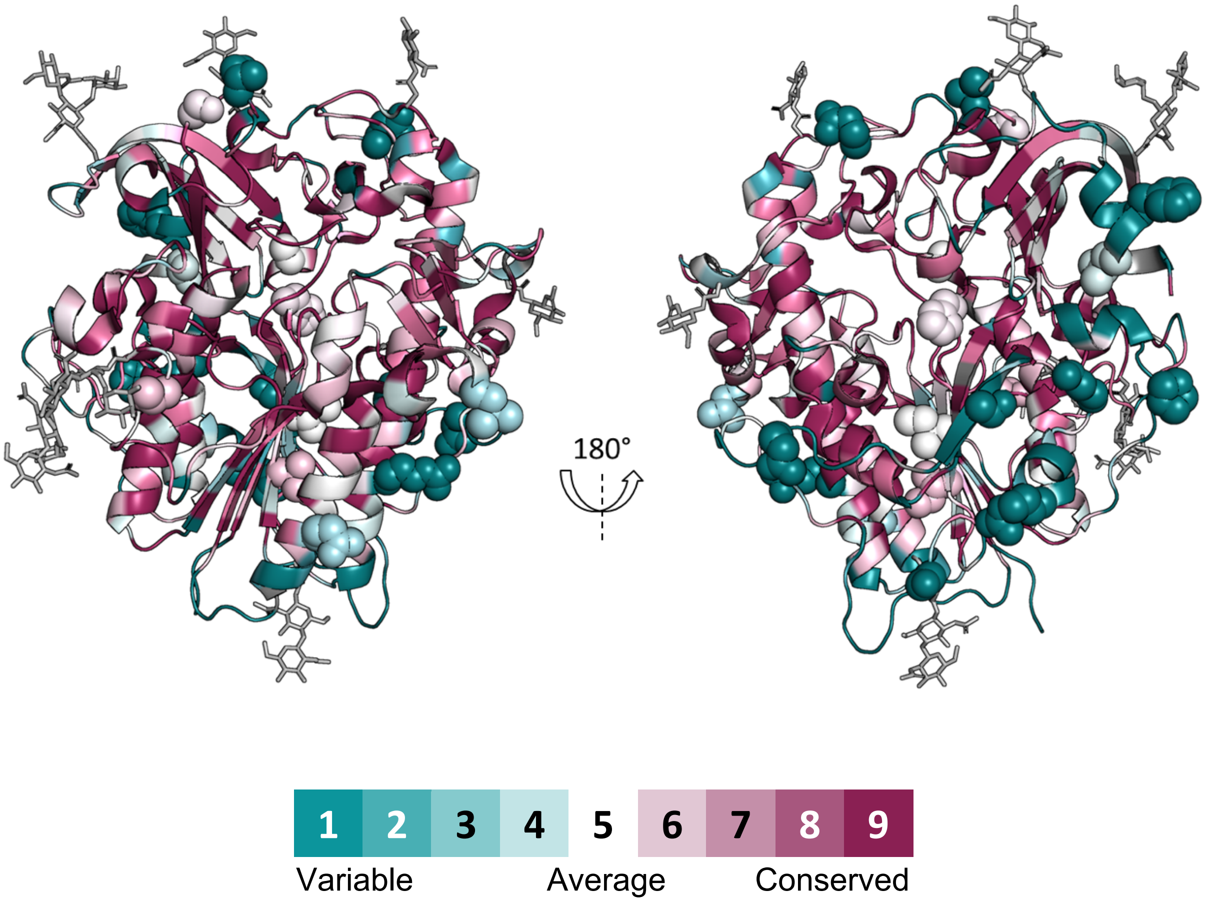

Figure S8: Analysis of evolutionary conservation in IDS alignment using ConSurf, related to Figure 1 and Figure 2. Conservation for each site in the IDS alignment that was used for ancestral reconstruction was analyzed using ConSurf. The structure of human IDS (PDB code: 5FQL (Demydchuk et al., 2017)) is shown (front view and back view) and colored according to conservation on a scale from 1-9, where 1 represents highly variable positions and 9 represents highly conserved positions. All 20 residues that are mutated in the ancestors are shown as spheres in their respective colors. Almost all ancestral mutations occur in positions with conservation score 1, apart from H356R (score: 4), T146S (score: 5) and I360 and S470 (score: 6). Glycans are shown as grey sticks.

Transparent Methods

Ancestral sequence reconstruction

A protein BLAST search was performed via NCBI in the non-redundant database using the sequence of hIDS (GenBank accession no. AAA16877.1) as a query. The 100 closest mammalian homologues were selected and duplicates, incomplete sequences, recombinant proteins and mutants were removed. The remaining 30 sequences were aligned in MAFFT version 7 (Katoh, 2002; Katoh and Standley, 2013) using the L-INS-i algorithm and were trimmed using trimAl (Capella-Gutiérrez et al., 2009) and the implemented "gappyout" method, which resulted in removal of approximately 10% of the positions. Model testing was performed in IQ-TREE (Nguyen et al., 2015) and MEGA7 (Kumar et al., 2016) and both found the Jones-Taylor-Thornton (JTT) model (Jones et al., 1992) to be the best evolutionary model for the dataset, including a gamma distribution (4 categories) to model rate variation across sites. The same model was found when performing the test with the full-length sequence alignment. A maximum likelihood tree was constructed in IQTREE with 1000 bootstrap replicates and the settings from the model test. We inferred the most probable ancestral sequences in PAML version 4 (Yang, 2007) at three different nodes, going back from hIDS to the last common ancestor of primates and rodents.

Homology modeling & MD simulations

A full-length model of hIDS was constructed using the homology modelling feature in ICM (Molsoft LLC) (Cardozo et al., 1995). The missing loop in the crystal structure (PDB: 5FQL (Demydchuk et al., 2017)) was added by sampling loop conformations from the PDB, followed by energy minimization. To allow for efficient MD analysis the fGly residue in position 84 was mutated to a serine, which has been shown to perform the same function as fGly in the coordination of the calcium ion in the related enzyme Arylsulfatase A, but rendering the enzyme inactive (von Bülow et al., 2001). A homology model for IDS-A3_T354A was built based on the full-length hIDS model by manually replacing the differing residues followed by energy minimization. Both models were subjected to MD simulations in the GROMACS software version 2018.4 (Pronk et al., 2013) using the Amber ff99SB-ILDN force field (Lindorff-Larsen et al., 2010). A cubic box was used for both systems with edges 1 nm from the protein, which was filled with SPC/E modeled water molecules (Kusalik and Svishchev, 1994). The systems were neutralized by adding 19 sodium ions, followed by energy minimization and equilibration with position restraints, a time step of 1 fs and divided in two phases; the first with an NVT ensemble for 100 ps, followed by an NPT ensemble for another 100 ps. Triplicate MD trajectories were run for each system for 100 ns at 300 K, taking the starting velocities from the NPT simulation and using no position restraints. Time steps of 2 fs were used and all run associated data were stored every 2 ps. The simulation used Particle-Mesh Ewald electrostatics (Darden et al., 1999), velocity-rescaling temperature coupling (Bussi et al., 2007) and Parrinello-Rahman pressure coupling (Parrinello and Rahman, 1981). The results were analyzed using GROMACS analytical tools.

Protein expression and purification

Sequences were codon-optimized for expression in *C. griseus*, synthesized by GeneArt (ThermoFisher Scientific) and subcloned into mammalian expression vector pcDNA3.4-TOPO. A C-terminal GGGSEPEA tag was included for affinity purification. ExpiCHO cells for transient expression (ThermoFisher Scientific) were transfected with vectors containing sequences of hIDS, mIDS, the hIDS variants and all ancestral enzymes (total plasmid concentrations of 1 µg/mL). The 50 mL cell cultures were treated according to the high titer protocol from the manufacturers manual and all reagents were from ThermoFisher Scientific. Cells were harvested 8 days after transfection by centrifugation for 30 min (4°C, 2264 x g). The supernatant was filtered through a 0.22 µm filter before being loaded onto a 1 ml CaptureSelect™ C-tagXL column (ThermoFisher Scientific) equilibrated with 20 mM Tris-HCl, 150 mM NaCl at pH 7.5, using an ÄKTA Explorer (GE Healthcare). Variants expressed without SUMF1 were

eluted with a 50 mM citrate, 150 mM NaCl buffer at pH 3.0 and elution fractions were analyzed by SDS-PAGE. Fractions containing the correctly sized protein were loaded onto a HiLoad 16/60 Superdex 200 column (GE Healthcare) which was equilibrated with a 20 mM Tris-HCl, 125 mM NaCl buffer at pH 7.5 and eluted with the same buffer. Elution fractions were analyzed on SDS-PAGE and fractions containing protein of the correct size were pooled, concentrated and stored at -80°C. Variants co-expressed with SUMF1 were eluted with a 20 mM Tris-HCl, 2 M $MgCl_2$ buffer at pH 7.1 and elution fractions were analyzed on SDS-PAGE. Fractions containing the correctly sized protein were loaded onto three 5 mL HiTrap Sephadex G-25 desalting columns (GE Healthcare) connected in tandem, which were equilibrated with a 20 mM Tris-HCl, 125 mM NaCl buffer at pH 7.5 and eluted with the same buffer. Elution fractions were analyzed by SDS-PAGE, and fractions containing protein of the correct size were pooled, concentrated and stored at -80°C.

Enzyme assays

IDS enzyme activity was measured in triplicates in a microplate format through a coupled reaction to **α**-L-iduronidase (Figure S1b) (Voznyi et al., 2001). All solutions and plates were pre-incubated at 37 °C and plates were sealed immediately after mixing the solutions. Reactions were initiated by mixing 10 µL IDS solution (1-2 nM, diluted with a diluent containing 0.2 % BSA, 3 mM NaN_3 and 0.05% Triton X-100) with 10 µL substrate solution (200 µM 4-MU-**α**IdoA2S and 0.1 mU **α**-L-iduronidase in a pH 4.5 buffer containing 0.1 M NaOAc in the diluent) in an incubation plate (PS96U) and were left shaking at 600 rpm at 37 °C in a microplate incubator (Thermostar, BMG Labtech). Blanks showed that 4-MU-**α**IdoA2S is not a substrate for **α**-L-iduronidase and they could be mixed before addition of IDS. The reaction was typically terminated after 35 min by addition of 100 µL stop buffer (40 mM $NaHCO_3$, 460 mM Na_2CO_3, 0.025% Triton X-100, pH 10.7). 100 µL of the terminated solution was then transferred to a reading plate (Black PS96F) and covered by a black lid before reading. The fluorescence (excitation: 355 nm, emission: 460 nm) of the solution in the well was measured by a multilabel plate reader (EnVision, PerkinElmer). The coupled reaction lag was determined to be 5 min under the assay condition. Product formation was calculated against a standard calibration curve of 4-MU, which was made in parallel with the assay wells at eight concentrations of 5 pmol interval from 0 to 35 pmol. One unit of IDS activity will hydrolyze 1.0 nmol of 4-MU-**α**IdoA2S per minute at pH 4.5, 37 °C under the defined condition.

Determination of formylglycine content

All reagents were purchased from Sigma-Aldrich unless stated otherwise and all mixtures were freshly prepared before the experiment. Enzyme samples (20 µg) were reduced, alkylated and digested with trypsin. Reduction was done by incubation in 5 µL DL-dithiothreitol (10 mM in 50 mM NH_4HCO_3) at 50 °C for 60 min. Subsequent alkylation with 5 µL iodoacetamide (55 mM in 50 mM NH_4HCO_3) was performed at room temperature in the dark for 45 min. For tryptic digestion 20 µL of 50 mM NH_4HCO_3, 5 mM $CaCl_2$ (pH 8) and trypsin (0.2 µg/µL sequencing grade, Promega) were added followed by incubation at 37 °C over night. The digestion was quenched by adding trifluoroacetic acid (TFA) to a final concentration of 0.5%. Prior to analysis 50 µL of 5% acetonitrile, 0.1% propionic acid and 0.02% TFA were added. Samples were transferred to HPLC vials and analyzed directly with liquid chromatography-mass spectrometry (LC-MS) (injection volume: 15 µL). LC separation was performed by use of a XSELECT CSH 130Å C18 column (100 x 2.1 mm, Waters) with a column temperature of 35 ^{0}C and a flow rate of 0.2 mL/min. Mobile phase A consisted of 5% acetonitrile, 0.1% propionic acid and 0.02% TFA and mobile phase B consisted of 95% acetonitrile, 0.1% propionic acid and 0.02% TFA. The gradient used was 0-5 min: 0-10% B, 5-30 min: 10-70% B, 30-35 min: 70-90% B, 35.1 min: 0% B. An Agilent 1200 HPLC system coupled to an Agilent 6545A Q-TOF-MS was used for the LC-MS analysis. During the course of data acquisition, the fragmentor voltage, skimmer voltage, and octopole RF were set to 175 V, 65 V, and 750 V, respectively. Scan range was set between 300 and 2800 m/z. The Q-TOF instrument was operated in positive electrospray ion mode and was controlled by MassHunter Workstation. Relative amounts of tryptic peptide SPNIDQLASHSLLFQNAFAQQAVCAPSR,

with the various modifications on Cys, were calculated by measuring the peak areas from reconstructed ion chromatograms of the triply charged ions. The following Cys variants were searched for: Cys (alkylated), FGly, Ser, hydroxylated Cys (sulfenic, sulfinic, sulfonic acid), Cys-SO_2-SH. No correction for ionization efficiency was done.

Thermostability measurements

Thermostability measurements were performed on a Prometheus NT.Plex nanoDSF instrument (NanoTemper Technologies) in a 20 mM Tris-HCl, 150 mM NaCl buffer at pH 7.5. Protein unfolding was monitored by following the ratio of intrinsic protein fluorescence at 350 nm to 330 nm over a temperature gradient from 20 °C to 90 °C with an increase of 1 °C per minute. The melting temperature corresponds to the maximum of the first derivative of the 350/330 ratio vs. temperature.

Analysis of cell uptake and intracellular activity

MPSII patient fibroblasts (GM00615, Coriell Institute) were seeded on 6-well plates at a density of 16000 cells/cm^2. One day after seeding, growth media was removed and treatment was added. The enzymes were diluted in growth media (+PEST) to a concentration of 2 nM and were further diluted to a series of six concentrations (2-0.01 nM for enzymes expressed without FGE and 0.2-0.001 for enzymes expressed with FGE), of which 2 mL was added to each well. The day after, cells were washed with cold PBS, trypsinized and resuspended in growth media. The mixtures were spun down and the cell pellets were washed in PBS. Cell pellets were resuspended in 100 µL 1 mM Tris (pH 7.6) for lysis, which was achieved by four freeze/thaw cycles. Debris was spun down and supernatants were saved for analysis of intracellular IDS concentration and substrate levels. The IDS concentration in the cell lysates were determined by an electrochemiluminiscence immunoassay using the Meso Scale Discovery (MSD) platform. The wells of a 96-well streptavidin gold MSD plate (#L155A-1) were blocked with 1% fish gelatin in PBS, washed with wash buffer (PBS + 0.05% Tween-20) and incubated with a biotinylated, affinity purified goat-anti-hIDS polyclonal antibody (BAF2449, R&D Systems) overnight in 4°C. After washing, different dilutions of standard and cell lysates in sample diluent (1% fish gelatin in wash buffer) were incubated in the plate shaking at 700 rpm in RT for 2 h. The plate was washed and an IDS-specific Rutenium (SULFO-TAG, MSD) tagged goat polyclonal antibody (AF2449, R&D) was added and allowed to bind to the captured IDS. The plate was washed and 2× Read Buffer (MSD) was added. The plate content was analyzed using an MSD Sector 2400 Imager Instrument.

In order to determine intracellular activity of the IDS enzymes the amount of sulfated trisaccharide substrate uronic acid – *N*-acetylglucosamine – uronic acid (UA-GlcNAc-UA(S)) was measured, which has previously been shown to be a marker for MPS (Fuller et al., 2004). Final concentrations of UA-GlcNAc-UA(S) were determined as follows: 0.1 ml 3-methyl-1-phenyl-2-pyrazolin-5-one (35 mg/ml in 0.4 M NH_3 pH 9.1 containing 47.5 % ethanol) and 20 µL of the internal standard chondroitin disaccharide Δdi-4S sodium (ΔUA-GalNAc4S, 10 µg/ml, Mw 459 Da) were added to the samples, followed by derivatization for 90 min at 70 °C. Samples were centrifuged for 6 min at 7000 rpm. The µ-WAX columns were preconditioned with 100 µl acetonitrile:10 M acetic acid (HOAc) (9:1 v:v) using low vacuum and the samples were transferred to the columns. The columns were washed with 0.2 mL methanol and eluted with 0.2 mL 0.4 M NH_3 in 60 % methanol into an LoBind 96-well plate (Eppendorf) using low vacuum. 20 µL of concentrated HOAc was added to all samples prior to injection (5 µl) into the LC-MS system. The samples were analyzed with LC–MS/MS using a QTrap 6500+ mass spectrometer equipped with an ESI interface. LC separation was performed on a Poroshell 120 EC-C18 column (2.7 µm, 50 × 2.1 mm, Agilent) with a flow rate of 0.45 ml/min. Mobile phase A consisted of 5% acetonitrile and 2% formic acid and mobile phase B consisted of 95% acetonitrile and 2% formic acid. The gradient program was equilibration of 95% A, followed by a linear decrease to 80% A (0.5 min), followed by another linear decrease to 75% A (1.7 min). The column was washed with 1% A (0.8 min) prior to re-equilibration with 95% A (3 min). Detection was carried out in negative ion mode using multiple reaction monitoring. The ionization optimization gave the following ESI settings: ion spray voltage 4.5 kV, curtain

gas 30 l/h, ion source gas1 60 l/h, ion source gas 2 60 l/h, and source temperature 500 °C. The precursor/product ions used for detection were internal standard Chondroitin disaccharide Δdi-4S (788.2/534.1) and UA-GlcNAc-UA(S) (490.5/476.0). The analytes elution times were between 1 and 3 min, the declustering potential used was -80 V and the collision energy was -18 eV. The data were acquired and processed using Analyst software (Sciex).

Analytical size exclusion chromatography

All purified enzymes were diluted to final concentrations of ca. 0.5-1.5 mg/ml in 20 mM Tris-HCl, 150 mM NaCl buffer (pH 7.5). Samples of 50 µL were loaded onto a Superdex 200 Increase 10/300 GL column equilibrated with phosphate-buffered saline (PBS, pH 7.4) and coupled to an ÄKTAmicro (GE Healthcare). Proteins were eluted using PBS (pH 7.4), and molecular weight of the proteins in the elution peaks was determined through online MALS detection, using a miniDAWN TriStar detector and ASTRA 5.3.4.20 software (Wyatt Technology).

Analysis of binding to mannose-6-phosphate receptor

Binding of IDS and its variants to the M6PR was compared by surface plasmon resonance using a Biacore T200 system (GE Healthcare). Recombinant human M6PR (R&D systems) was immobilized on a CM5 sensor chip (GE Healthcare) using an amine coupling kit (GE Healthcare) to approximately 8000 RU. IDS enzymes were diluted to a final concentration of 1 µM in running buffer (10 mM HEPES, 150 mM NaCl, 0.05 % P20, pH 7.4) and injected at 30 µl/min. Measurements were performed using the single cycle kinetics mode with an injection time of 180 s and a dissociation time of 180 s. The sensor surface was regenerated for 90 s + 30 s with regeneration buffer (10 mM NaOAc, 300 mM NaCl, pH 5.0). Results were evaluated using the Biacore T200 evaluation software v3.0 (GE Healthcare).

Analysis of glycans

An analysis of the glycans of different IDS batches was performed using the GlycoWorks *Rapi*Fluor-MS N-Glycan Kit (Waters). Enzymes were concentrated to final concentrations of 1-2 mg/mL and 15 µg of enzyme was used for the assay. The enzymes were denaturated at 90 °C for 5 min followed by deglycosylation using PNGase F (Promega, #V4831) at 37°C over night. Finally, the enzymes were labelled according to the manufacturer's instructions. Samples were analyzed on a LC-FLR-MS QToF system (Agilent), using a Waters Glycan BEH Amide column (2.1x150 mm, 130 Å 1.7 µm) with a column temperature of 52 °C and using the gradient and flow recommended in the GlycoWorks *Rapi*Fluor-MS N-Glycan protocol.

Supplemental References

von Bülow, R., Schmidt, B., Dierks, T., von Figura, K., and Usón, I. (2001). Crystal Structure of an Enzyme-Substrate Complex Provides Insight into the Interaction between Human Arylsulfatase A and its Substrates During Catalysis. J Mol Biol *305*, 269–277.

Bussi, G., Donadio, D., and Parrinello, M. (2007). Canonical sampling through velocity rescaling. J Chem Phys *126*, 14101–14107.

Capella-Gutiérrez, S., Silla-Martínez, J.M., and Gabaldón, T. (2009). trimAl: A tool for automated alignment trimming in large-scale phylogenetic analyses. Bioinformatics *25*, 1972–1973.

Cardozo, T., Totrov, M., and Abagyan, R. (1995). Homology modeling by the ICM method. Proteins Struct. Funct. Bioinforma. *23*, 403–414.

Darden, T., Perera, L., Li, L., and Pedersen, L. (1999). New tricks for modelers from the crystallography toolkit: the particle mesh Ewald algorithm and its use in nucleic acid simulations. Structure *7*, 55–60.

Fuller, M., Rozaklis, T., Ramsay, S.L., Hopwood, J.J., and Meikle, P.J. (2004). Disease-specific markers for the mucopolysaccharidoses. Pediatr. Res. *56*, 733–738.

Jones, D.T., Taylor, W.R., and Thornton, J.M. (1992). The rapid generation of mutation data matrices from protein sequences. Bioinformatics *8*, 275–282.

Katoh, K. (2002). MAFFT: a novel method for rapid multiple sequence alignment based on fast Fourier transform. Nucleic Acids Res. *30*, 3059–3066.

Katoh, K., and Standley, D.M. (2013). MAFFT multiple sequence alignment software version 7: Improvements in performance and usability. Mol. Biol. Evol. *30*, 772–780.

Kumar, S., Stecher, G., and Tamura, K. (2016). MEGA7: Molecular Evolutionary Genetics Analysis version 7.0 for bigger datasets. Mol. Biol. Evol. *33*, 1870–1874.

Kusalik, P.G., and Svishchev, I.M. (1994). The Spatial Structure in Liquid Water. Science *265*, 1219–1221.

Lindorff-Larsen, K., Piana, S., Palmo, K., Maragakis, P., Klepeis, J.L., Dror, R.O., and Shaw, D.E. (2010). Improved side-chain torsion potentials for the Amber ff99SB protein force field. Proteins *78*, 1950–1958.

Nguyen, L.T., Schmidt, H.A., Von Haeseler, A., and Minh, B.Q. (2015). IQ-TREE: A fast and effective stochastic algorithm for estimating maximum-likelihood phylogenies. Mol. Biol. Evol. *32*, 268–274.

Parrinello, M., and Rahman, A. (1981). Polymorphic transitions in single crystals: A new molecular dynamics method. J. Appl. Phys. *52*, 7182–7190.

Pronk, S., Páll, S., Schulz, R., Larsson, P., Bjelkmar, P., Apostolov, R., Shirts, M.R., Smith, J.C., Kasson, P.M., van der Spoel, D., et al. (2013). GROMACS 4.5: a high-throughput and highly parallel open source molecular simulation toolkit. Bioinformatics *29*, 845–854.
